# Supplementary figures and images for: Comprehensive analysis of cancer breakpoints reveals signatures of genetic and epigenetic contribution to cancer genome rearrangements
Source: PLoS Comput Biol. 2021 Mar 1;17(3):e1008749. doi: 10.1371/journal.pcbi.1008749 (PMC7951985; doi:10.1371/journal.pcbi.1008749)

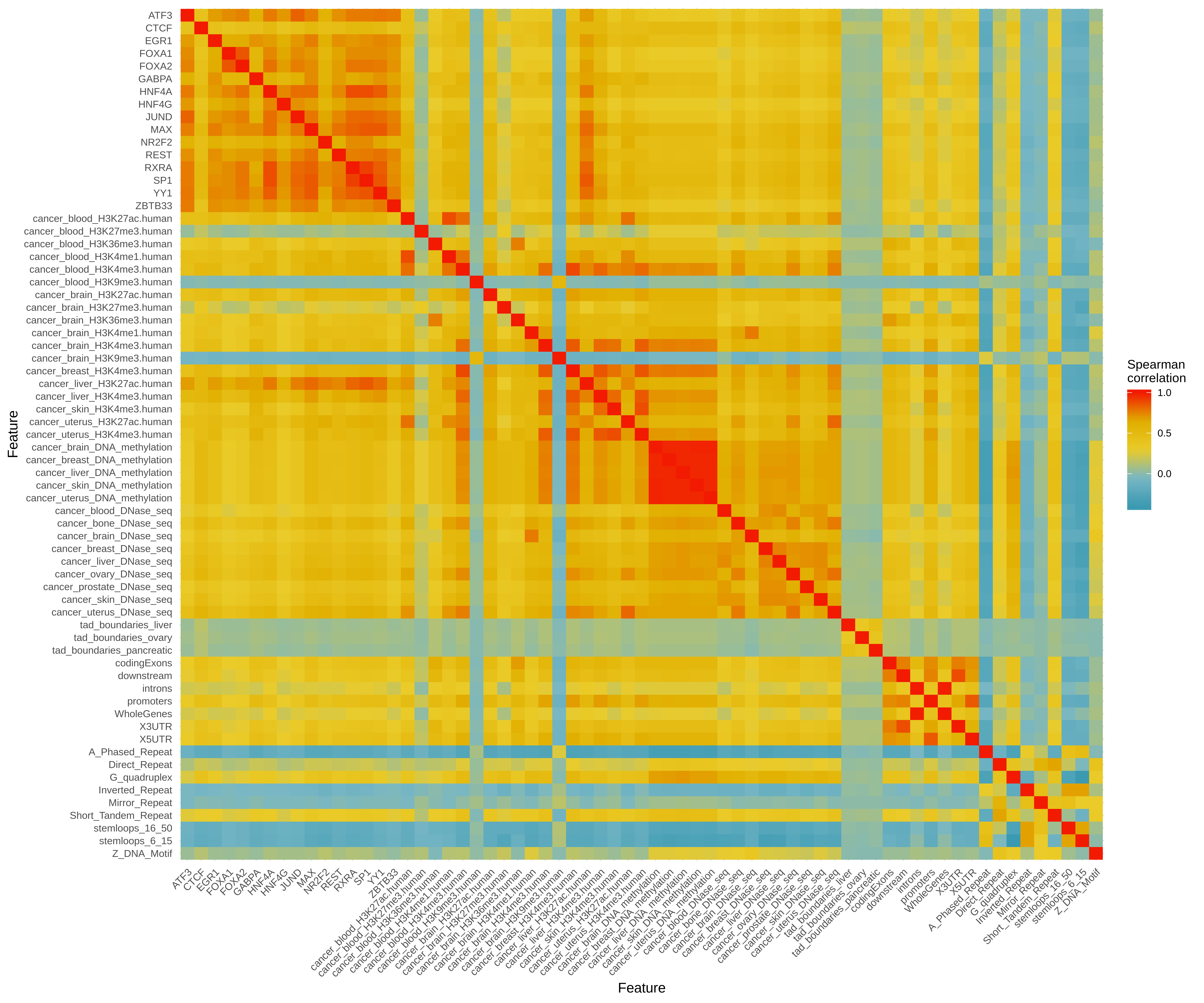

Supplement: S1 Fig — (TIFF) [file pcbi.1008749.s001.tiff]

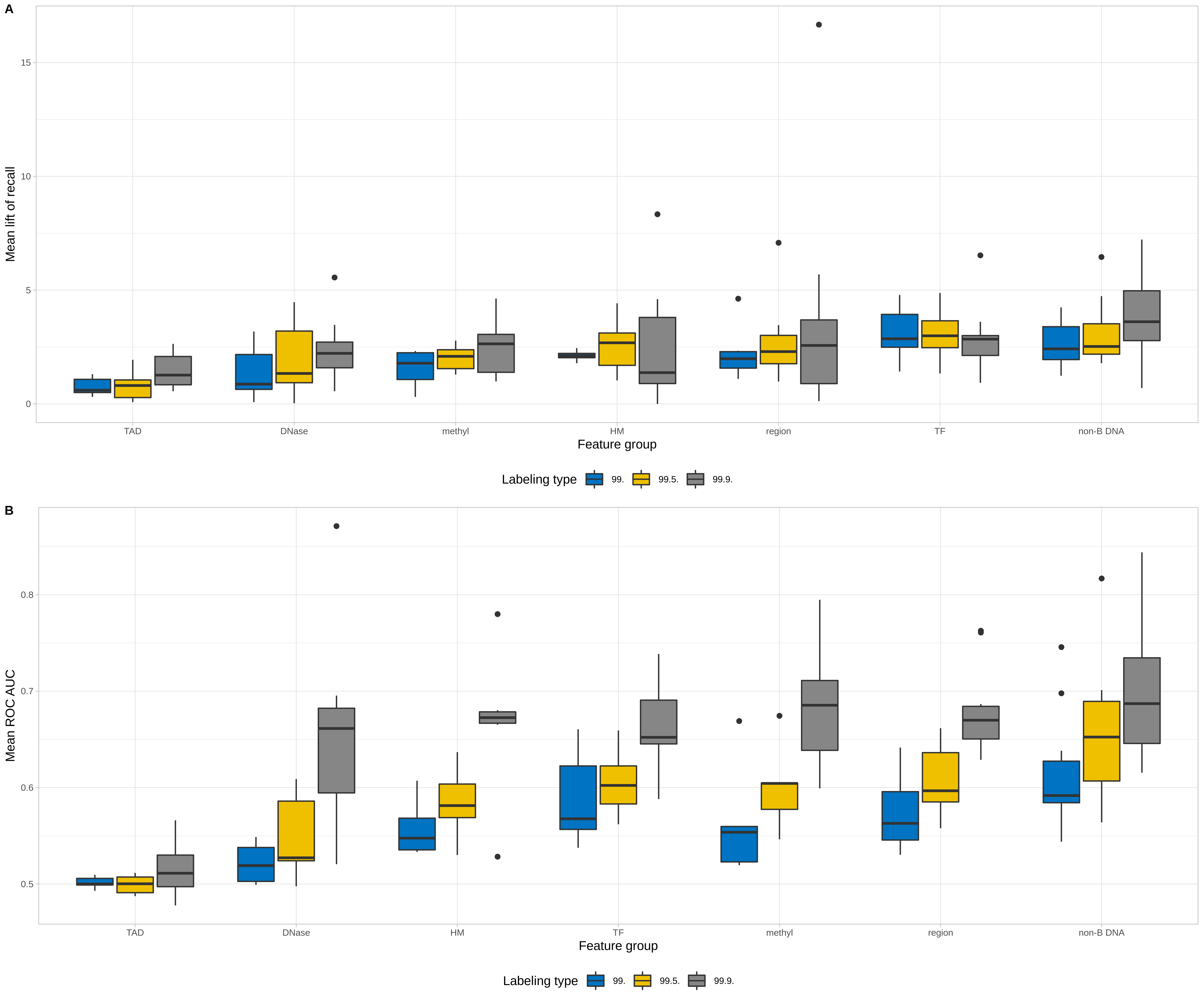

Supplement: S2 Fig — A. Distribution of mean lift of recall for 0.03 probability percentile by feature group and labelling type over all cancer types. B. Distribution of mean ROC AUC by feature group and labelling type over all cancer types. (TIFF) [file pcbi.1008749.s002.tiff]

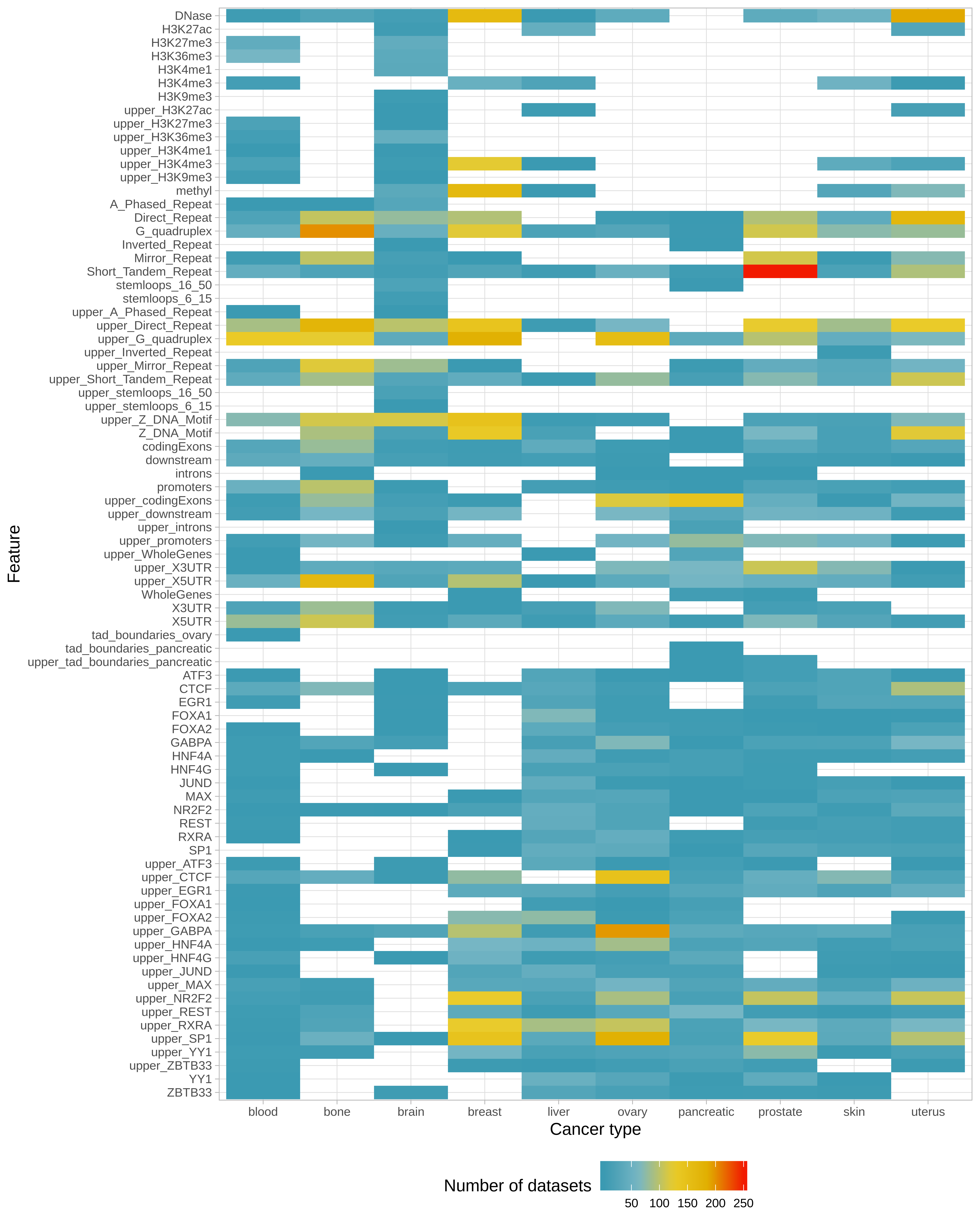

Supplement: S6 Fig — Number of executions in which a feature was recognized as important by Boruta feature selection in hotspots prediction models. (TIFF) [file pcbi.1008749.s006.tiff]

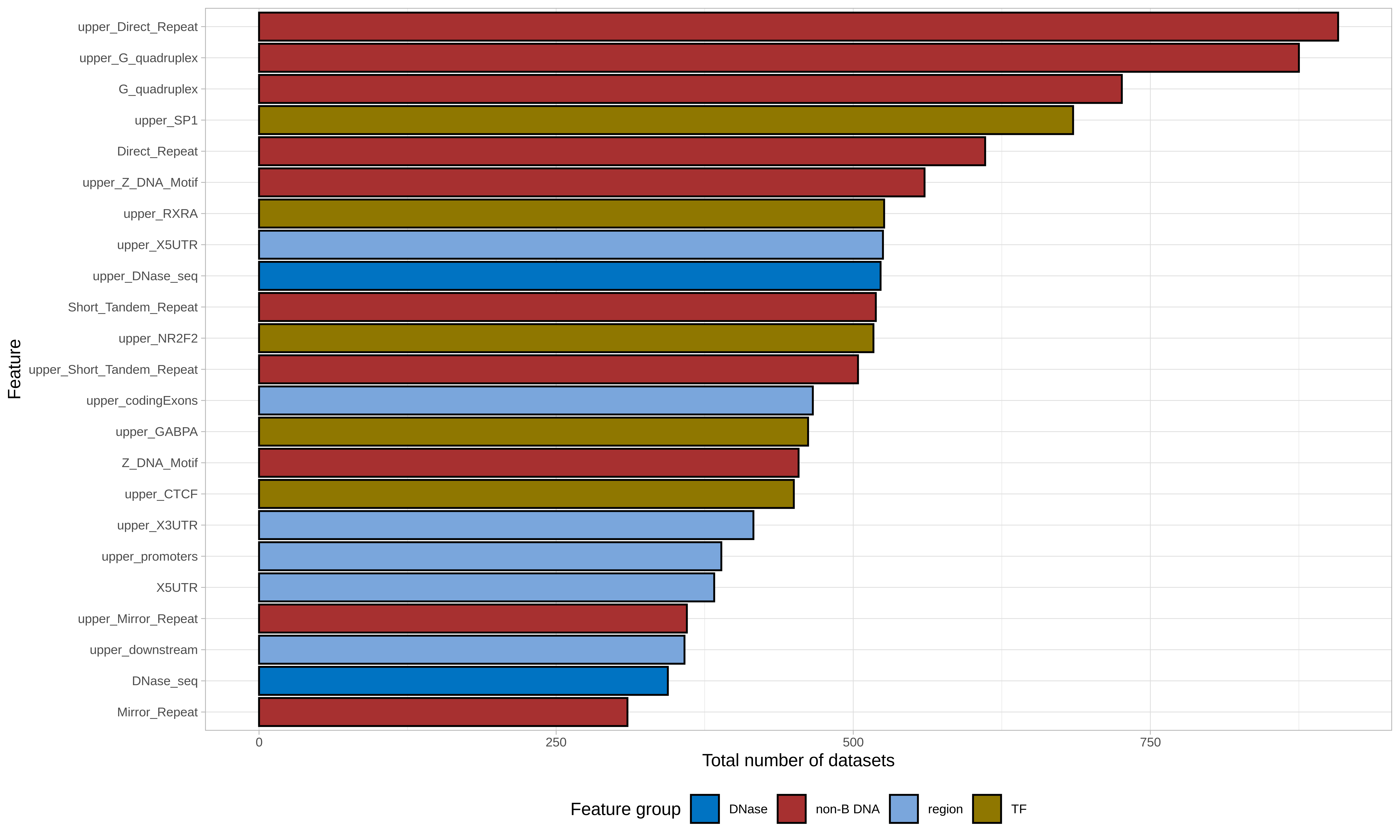

Supplement: S7 Fig — The number of datasets for which feature was considered as important in Boruta feature selection procedure for all cancer types. Only features with the total number of datasets no less than 300 are included. (TIFF) [file pcbi.1008749.s007.tiff]

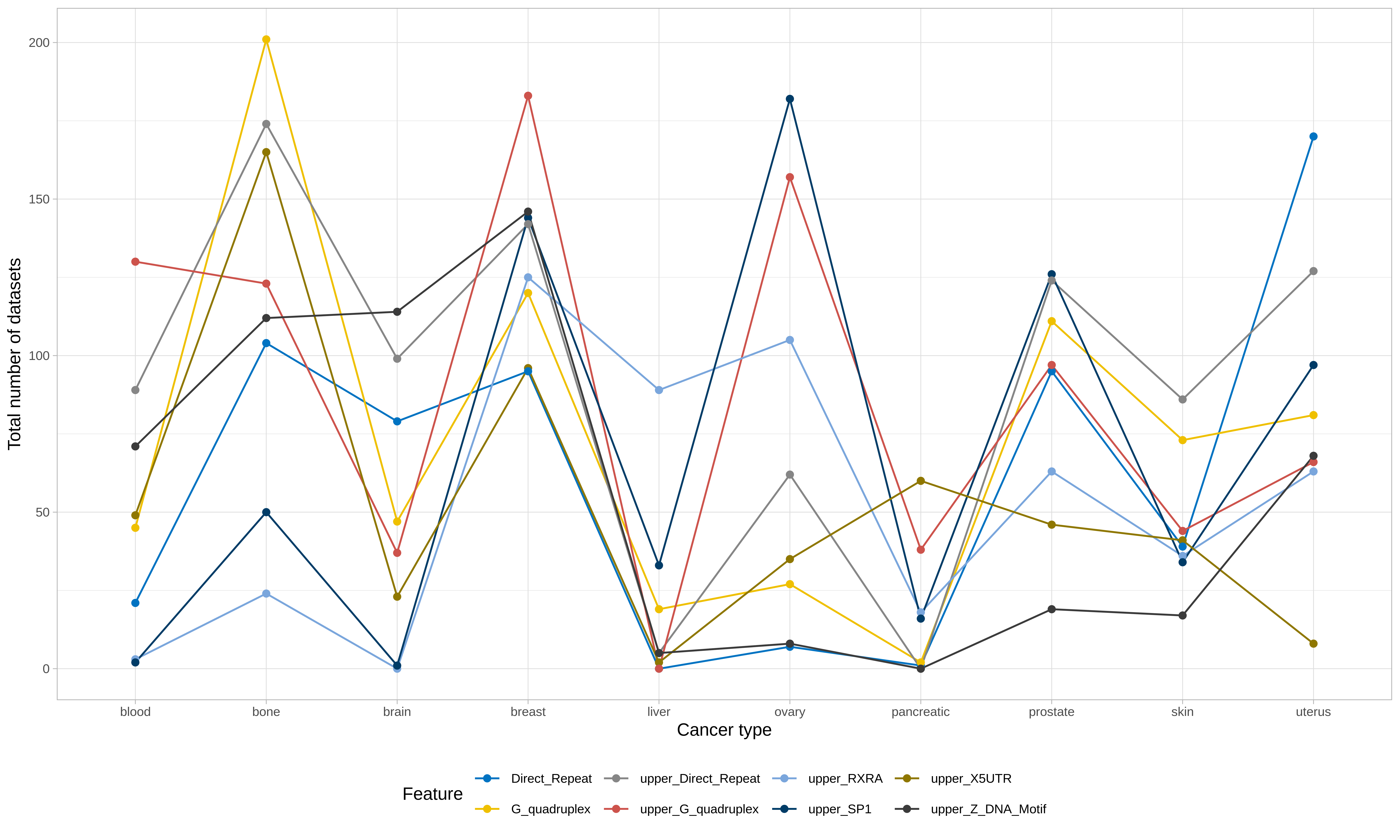

Supplement: S8 Fig — (TIFF) [file pcbi.1008749.s008.tiff]

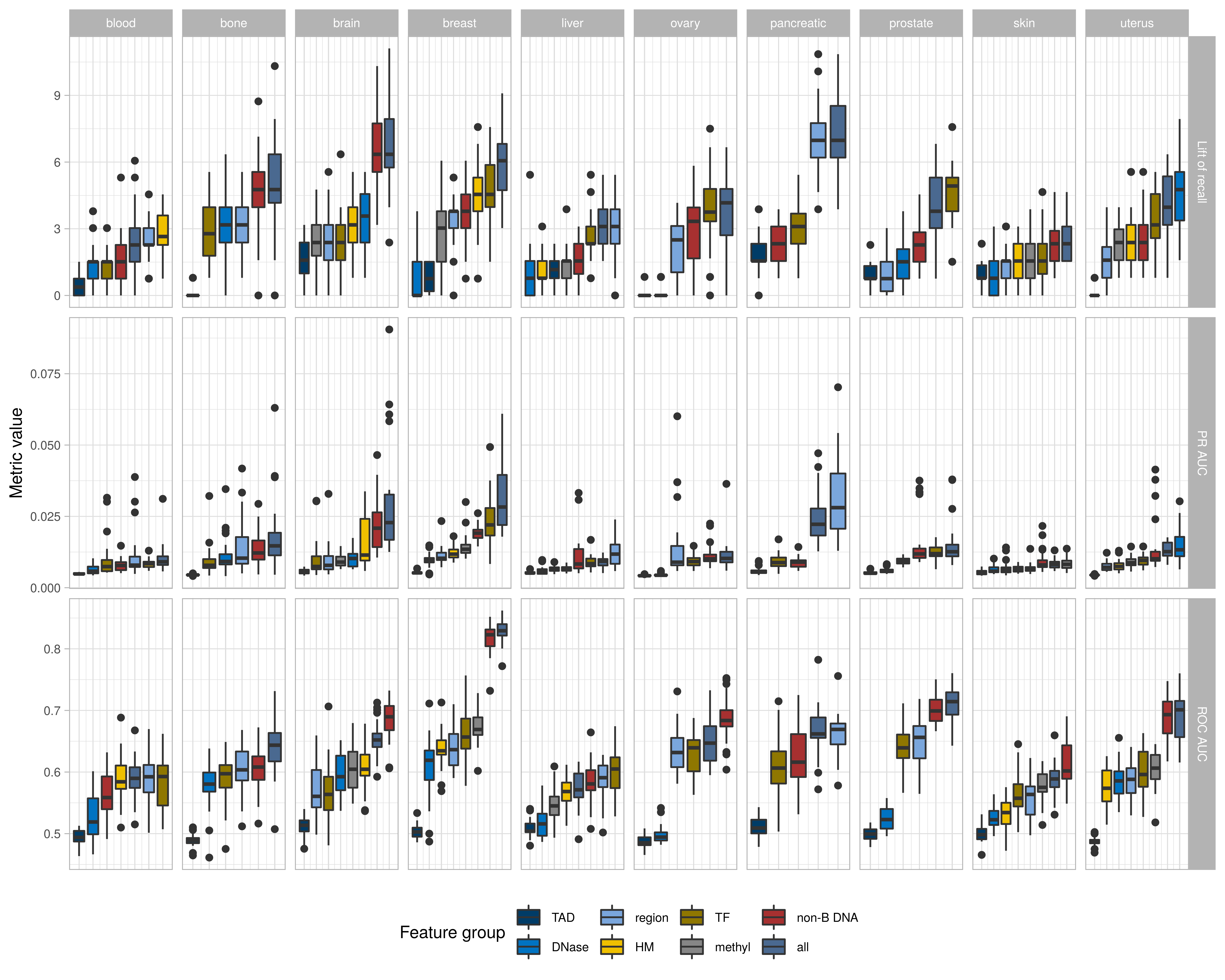

Supplement: S9 Fig — Distribution of lift of recall for 0.03 probability percentile, PR AUC and ROC AUC for each of one group-based models and models in Boruta selected features (for 99.5% labeling type). (TIFF) [file pcbi.1008749.s009.tiff]

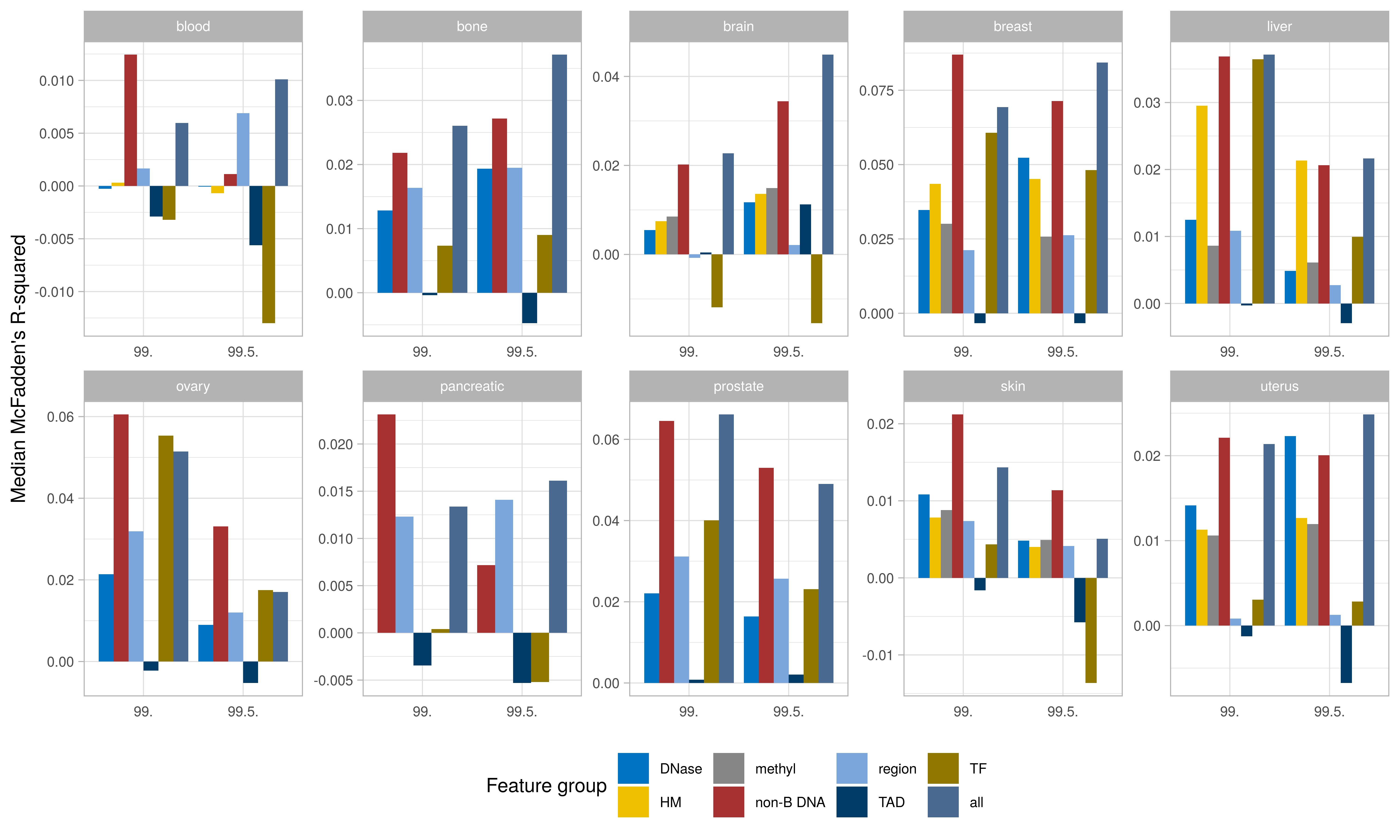

Supplement: S10 Fig — Median values of McFadden’s R-squared for modelling hotspots (99% and 99.5% labelling type) with logistic regression. (TIFF) [file pcbi.1008749.s010.tiff]

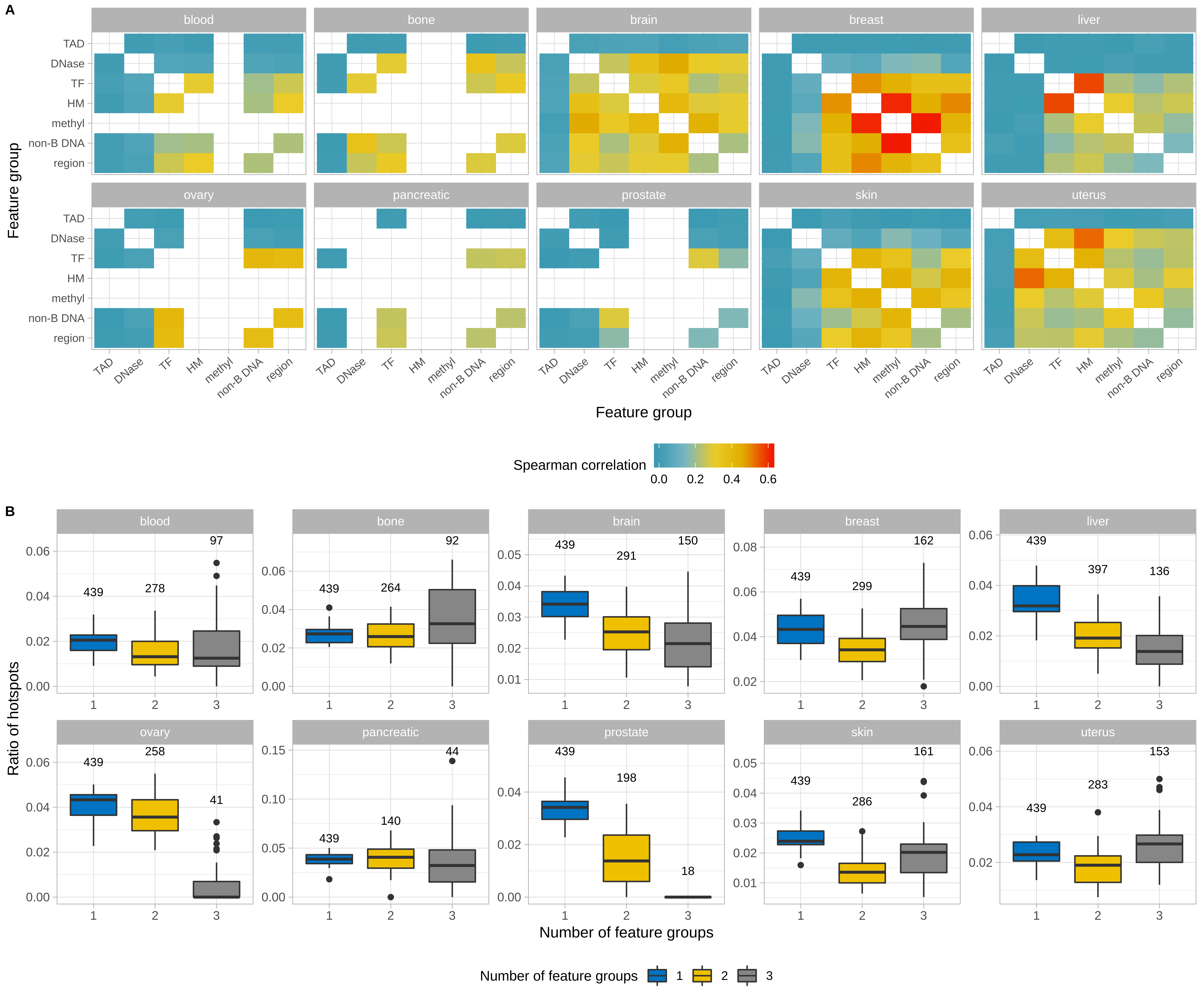

Supplement: S11 Fig — A. Mean Spearman correlation for predicted probabilities of genome window being a hotspot for different feature group based models for 99% labeling type. B. Distribution of precision at threshold corresponding to selection of 5% of genome windows with the highest probability for 99% labeling type. At x-axis the number of intersected feature group based models predictions is depicted (label "1" corresponds to best single feature group based model predictions while label "2" denotes the genome windows where exactly two feature group based models predictions intersected). Annotation—number of selected genome windows marked as hotspots. (TIFF) [file pcbi.1008749.s011.tiff]

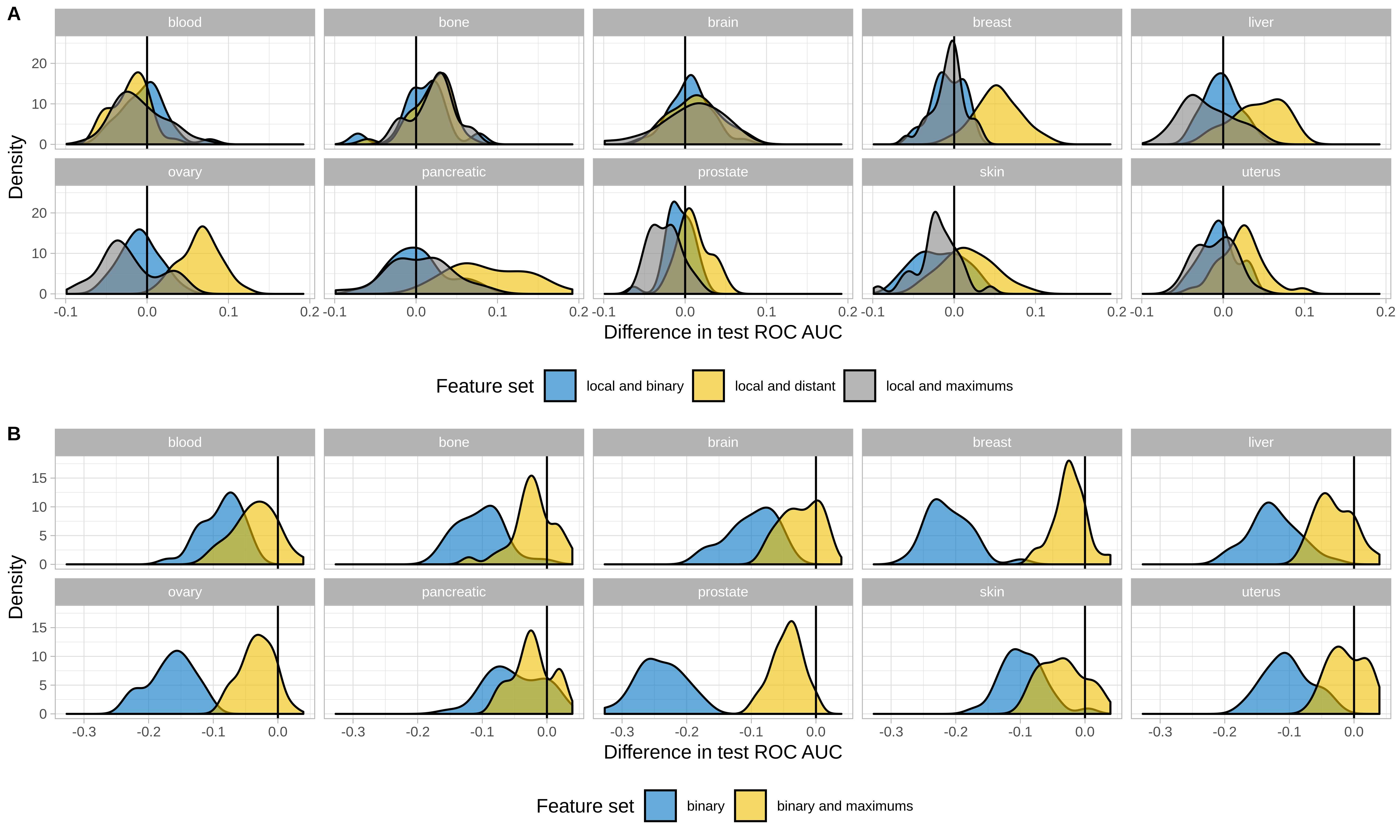

Supplement: S12 Fig — A. Distribution of difference between test ROC AUC of model on extended feature sets and on local features. B. Distribution of difference between test ROC AUC of model on binary features / indicators of maximums and on local features. (TIFF) [file pcbi.1008749.s012.tiff]

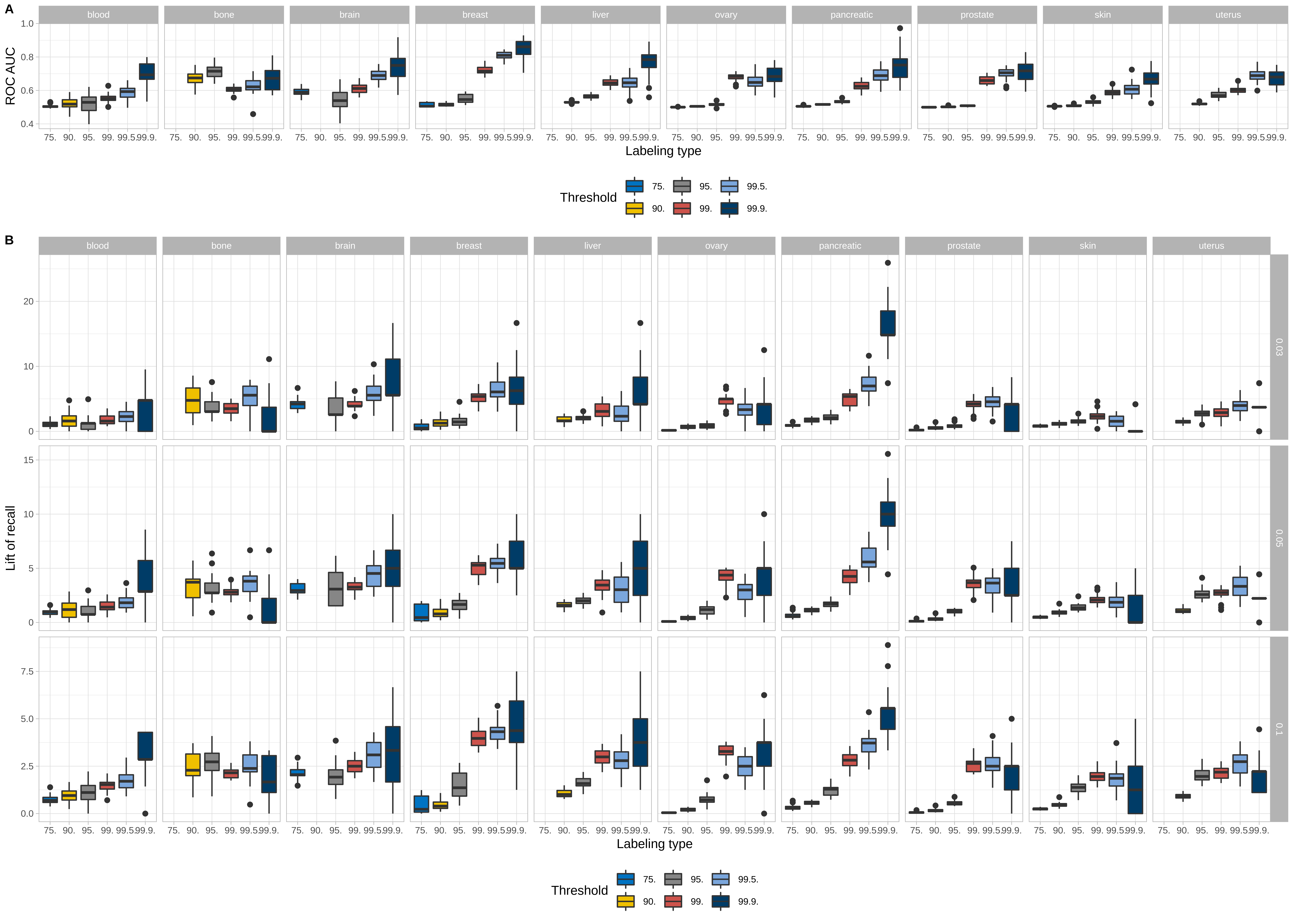

Supplement: S13 Fig — Distribution of test ROC AUC and lift of recall for 0.03, 0.05 and 0.1 probability percentiles for hotspots prediction models with high and low hotspots labeling thresholds for each type of cancer. (TIFF) [file pcbi.1008749.s013.tiff]

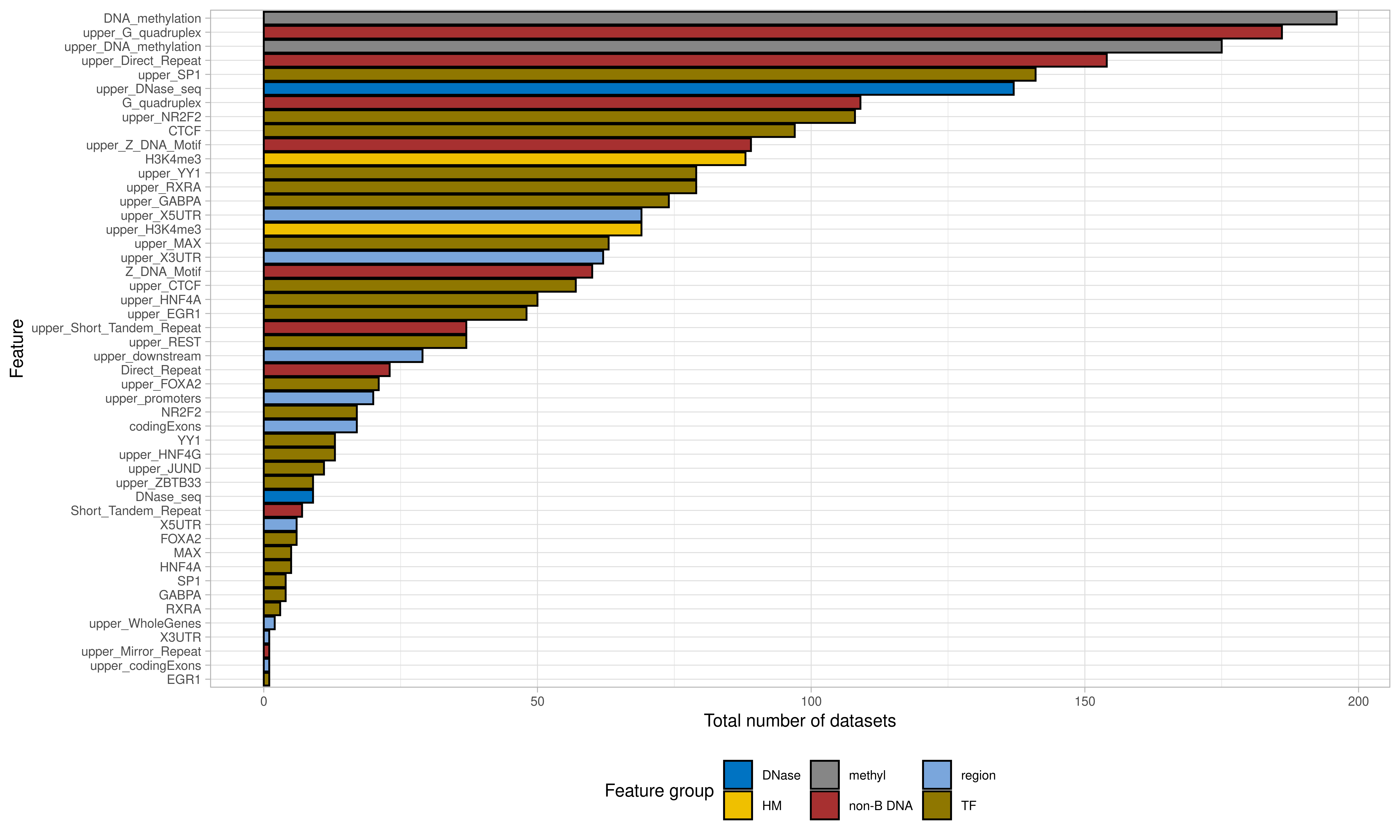

Supplement: S14 Fig — The number of datasets for which feature was considered as important in Boruta feature selection procedure for breast cancer (99% labelling type). (TIFF) [file pcbi.1008749.s014.tiff]

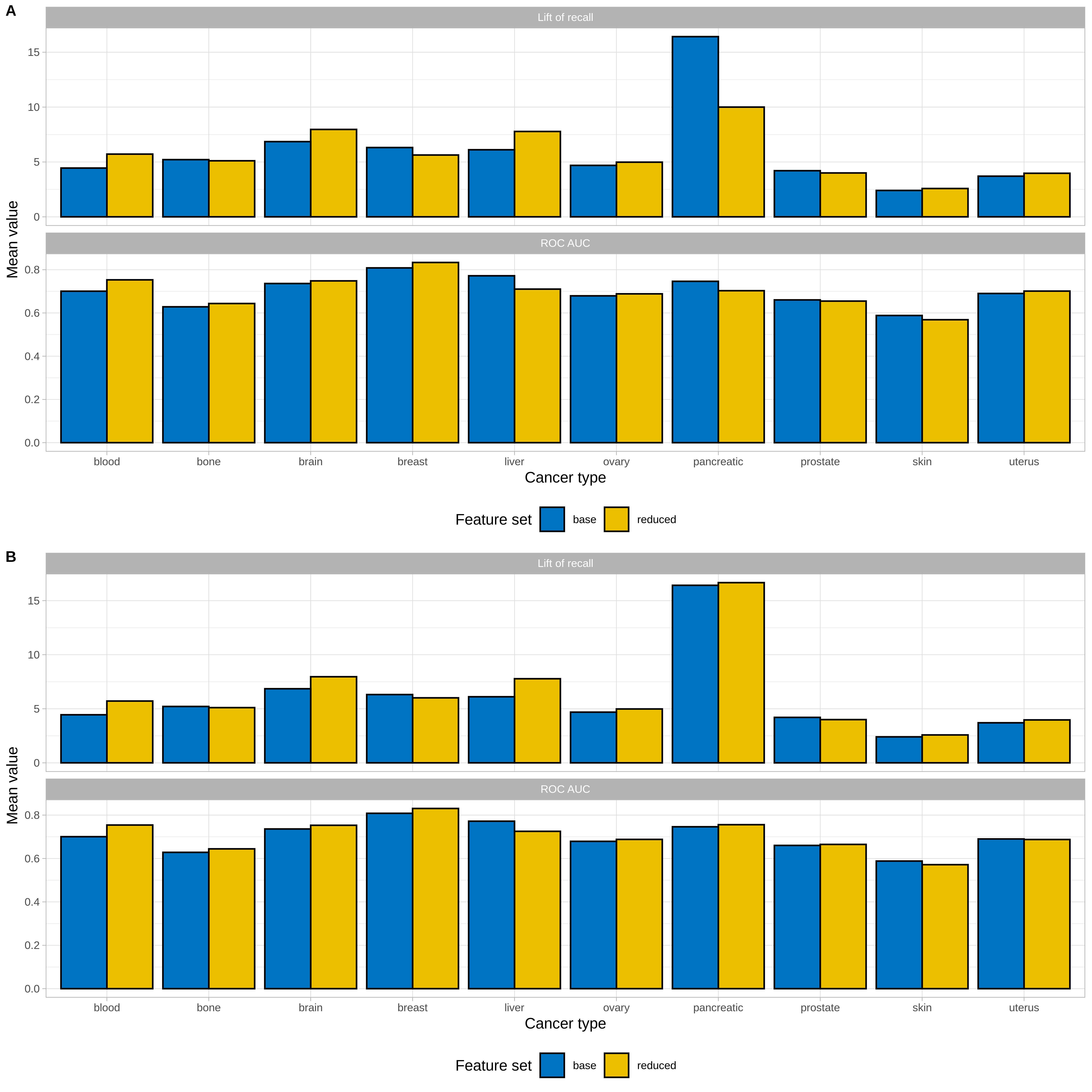

Supplement: S16 Fig — A. Comparison of mean test lift of recall and mean ROC AUC for hotspots prediction models trained on all features and on reduced sets of features (the best feature sets produced by Boruta feature selection) for 0.03 probability percentile threshold. B. Comparison of mean test lift of recall and mean ROC AUC for hotspots prediction models trained on all features and on reduced sets of features (the best feature sets produced by Boruta feature selection extended with several important features) for 0.03 probability percentile threshold. (TIFF) [file pcbi.1008749.s016.tiff]

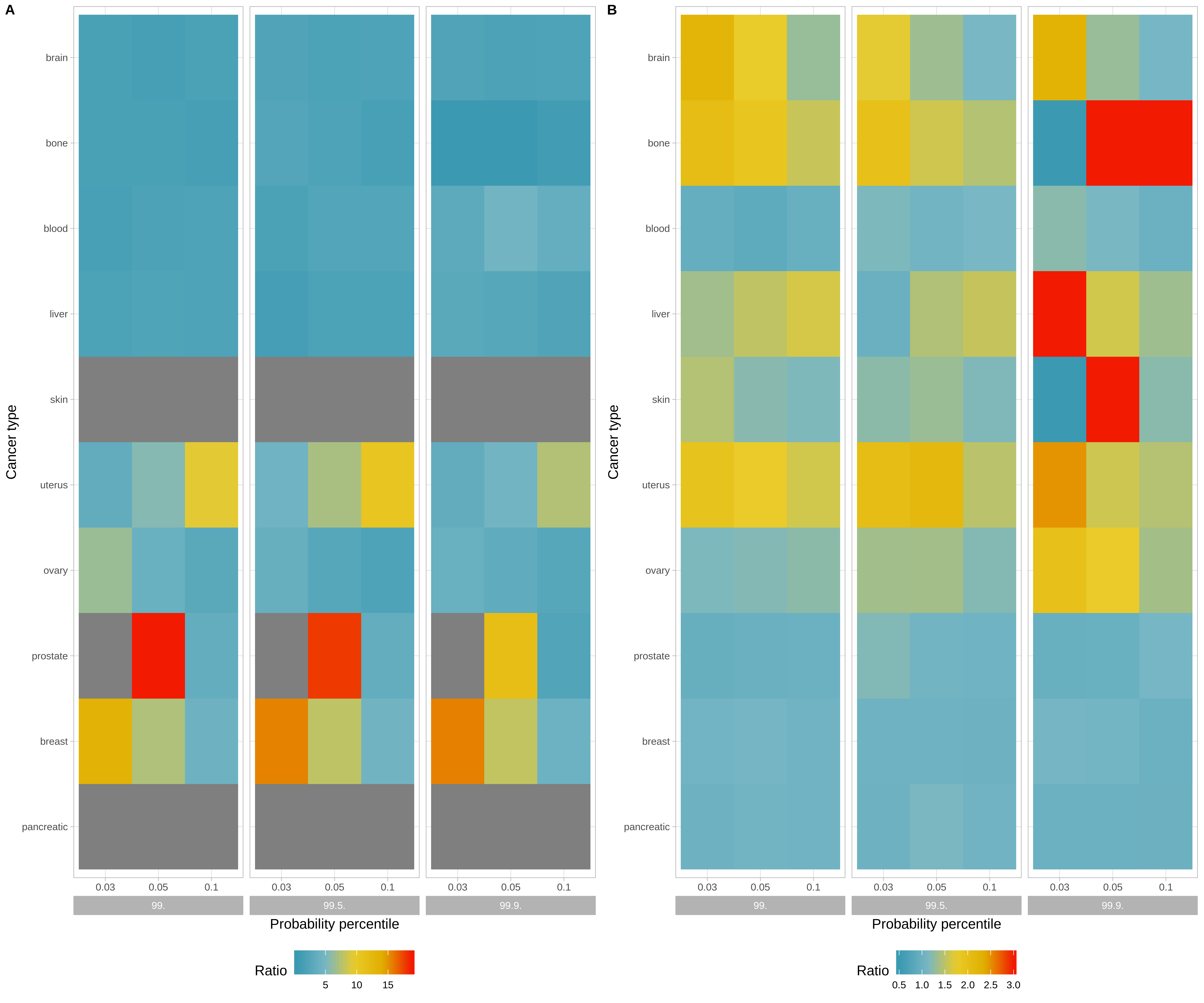

Supplement: S17 Fig — A. Ratio of lower bound of confidence interval for mean lift of recall for "hotspots vs all" prediction model to median lift of recall for "breakpoints vs all" prediction model at 0.03, 0.05 and 0.1 probability percentile. Gray colour corresponds to infinity value meaning that is equal to zero. B. Ratio of lower bound of confidence interval for mean lift of recall for "hotspots vs all" prediction model to lower bound of confidence interval for mean lift of recall for "hotspots vs breakpoints" prediction model at 0.03, 0.05 and 0.1 probability percentile. For visualization purposes two negative outliers (-27.82 and -1.05) were set to 0.5 and 4 positive outliers (from 3,39 to 8,12) were set to 3. (TIFF) [file pcbi.1008749.s017.tiff]
