# Supplementary material for: Comprehensive analysis of cancer breakpoints reveals signatures of genetic and epigenetic contribution to cancer genome rearrangements
Source: PLoS Comput Biol. 2021 Mar 1;17(3):e1008749. doi: 10.1371/journal.pcbi.1008749 (PMC7951985; doi:10.1371/journal.pcbi.1008749)
Supplement: S1 File — (PDF) [file pcbi.1008749.s039.pdf]

Supplementary Methods

Data

We used data of 628,126 breakpoints of cancer genomes from 10 types of cancer (release 28 from ICGC[1]). Compared to previous analysis [2] that used earlier data release this data set was enlarged by more than 165,000 new breakpoints, which became available mostly for prostate cancer (more than 128,000 new breakpoints) (Supplemental Table S2). Breakpoints data were pre-processed prior to the analysis. First, the data were filtered so that breakpoints with a high inaccuracy in the position (start/end location interval is wider than 10 bases) were removed from the sample (Supplemental Table S3). Then the genome was split into non-overlapping windows of 100 kb width.

We excluded windows intersected with centromeres and telomeres, and the first and last window of each chromosome. We removed region from the DAC blacklist (https://www.encodeproject.org/annotations/ENCSR636HFF/) comprising regions of low mappability. Concerning blacklisted regions the percent of breakpoints overlapping with these regions is very small. So it is minimal for liver cancer (0.09%), maximal for brain cancer (6,8%) and it is in the range of 0.4% - 1.5% for other cancer types. Y and MT chromosomes were not included in the study as they contain relatively small number of breakpoints. Finally we selected 29288 100-kb windows.

For each window, located in a specific chromosome, the breakpoint density was calculated as the number of breakpoints overlapping with the window divided by the total number of breakpoints in the chromosome separately for each cancer type. Given the breakpoint density we highlighted hotspots - regions of the genome significantly enriched with breakpoints. Three percentiles - 99%, 99.5% and 99.9% - of breakpoint density distribution were calculated for each cancer type and used as a threshold for labeling hotspots. The total number of breakpoints sorted by cancer type and labeling is given in Supplemental Table S4. Additionally, binary labels were assigned to genome windows indicating presence/absence of breakpoints.

Genomic feature data were downloaded from several projects such as the ENCODE [3, 4], Non-B DB [5], UCSC Genome Browser. Non-B DNA structure annotations for all types except for stem-loops were collected from Non-B DB while the

latter were provided by the DNA punctuation project. Genomic regions annotations were downloaded from UCSC Genome Browser. Topologically associating domain (TAD) data were taken from [6] (Table S3 “TAD boundary annotations”). The epigenetic features data were downloaded from the ENCODE. We selected experiments with the status “released”, the number of biological replicates greater than one and without any treatment therapy. For transcription factors we used TF ChIP-seq, for DNA accessibility - DNase-seq, for histone modifications - Histone ChIP-seq, for DNA methylation - RRBS.

Feature data were aggregated in chromosome windows by calculating the coverage as the summed length of all regions in a window covered by a feature (avoiding double count of overlapping regions) divided by the window length so that the coverage reflects how many bases in a window are covered by the feature.

Model selection (hyperparameters search)

Three labeling types for each of 10 types of cancer comprised 30 datasets. To get reliable model performance estimates we used 30-times repeated stratified train-test split with 30% of data in a test sample with splits stratified by a chromosome and position quarter inside it. The algorithm of choice was Random Forest.

As the selected labeling types imply strong class imbalance, we used specific oversampling technique. If the current class balance (the ratio of positive examples) is less than 0.2 then the minority class examples are all retained while the majority class is sampled so that the final class balance will be restored. Alternatively, if the current class balance is higher than 0.5 (so that there are more positive than negative examples) then it will be made equal to 0.5 by sampling positives. Otherwise the balance is considered as natural and no changes are made. This class balancing technique is used when defining sample size for building each tree in Random Forest.

At this point we checked two hypotheses: whether it is required to winsorize a feature vector and what are optimal hyperparameters for the selected algorithm. To test the first hypotheses we used the data of 99% labeling type as the most stable type. We selected examples (genome windows), which have negative hotspot labels for all cancer types and fixed 30 train-test splits of this set. Then for each cancer type positive examples were also split and added to the training and testing sets. This method of resampling allowed us choosing the best hyperparameters. Further, using class balancing technique we performed grid search on the given datasets with and without

winsorizing. Cancer types with minimal test ROC AUC higher than 0.55 were taken into account. For each cancer type and the selected hyperparameters, we calculated the standard deviation of test ROC AUC, mean and median difference between train and test ROC AUC. For each cancer type the distribution of test ROC AUC standard deviation was split into 5 bins with an equal number of observations in each. Next for each cancer type and hyperparameters the statistics was replaced with the bin number it belongs to. Then for each cancer type and set of hyperparameters these metrics values were ranked in the descending order (the lower rank, the better statistic value). In the end, we determined the optimal set of hyperparameters as the set with the minimal sum averaged over all cancer types metrics ranks.

Our experiments showed that model performance is higher without winsorizing. The following values of hyperparameters for Random Forest model were chosen: maximal number of terminal nodes - 3, number of trees - 500, minimal number of observations in a terminal node - 30, number of sampled variables - 5. Yeo-Johnson transformation, centering and scaling were applied to features before training the model.

Feature engineering

We checked whether different aggregates or transformations of features can improve model performance.

For each original feature represented by coverage (referred to as local feature) the following new features were created:

1. Binary feature of presence (1, if coverage of the genomic characteristic is greater than 0, and 0 otherwise)
2. Indicators of local/global maximums (binary indicator of the feature value exceeding 90%/ 95%/ 99% percentiles of the feature distribution; binary indicator of the feature value exceeding values of all 1/5/10 previous and following neighbours; relative difference between the feature value and maximum of 10 previous and following neighbours).
3. Distant features (coverage of the feature on 1Mb window)

Models with engineered features were constructed for hotspots of 99% labeling type.

The following feature sets were tested:

- local features and binary features of presence
- local features and features of local/global maximums

- local features and distant features

The quality of models constructed using engineered features was compared to the quality of models for corresponding cancer types built on the local features. Distribution of difference between test ROC AUC of models using extended feature sets and base feature set is given in Supplemental Figure 11A. According to the obtained results, only distant features were selected to be included in final feature set.

Evaluation metrics

To evaluate the performance of the machine learning models such metrics as ROC AUC, PR AUC, recall and lift of recall, precision and lift of precision were used.

The lift of recall for a specific probability threshold (given by the percentile of the probability distribution) is calculated as the recall divided by this percentile of the probability distribution. Since for a random choice this proportion is approximately equal to 1 (labeling n% of examples gives n% recall), the lift of recall for a model shows the number of times the model performs better than a random choice. Similarly, the lift of precision is determined as the precision divided by the proportion of positive examples in a dataset and demonstrates the number of times a model performs better than a random choice.

In this research lifts of metrics are evaluated rather than metrics as they make it possible to compare models trained on datasets with different class balance.

Breakpoints hotspots model evaluation

Having an optimized feature set and model specification we built machine-learning models to predict cancer breakpoints hotspots of three labeling types as well as individual cancer breakpoints. These models were compared in terms of ROC AUC, lift of recall and lift of precision.

First, we analysed the dependence of hotspots prediction model quality on labeling types. The considered metric distributions are presented in Supplemental Figure 13. Obviously, there is no single labeling type that could be selected as the most performant for all cancer types. Hence, it is required to select the best hotspot labeling type separately for each type of cancer. The choice was made based on the lift of recall and lift of precision; the summary of the performance metrics for the best labeling type is given in Supplemental Table S12.

It could be seen that for 4 cancer types - brain, breast, liver, pancreatic – the median test ROC AUC higher than 0.7 is achieved with maximum for breast cancer (0.81). In contrast, the minimal median test ROC AUC is observed for skin (0.59) and bone cancer (0.62). Besides, the minimal test ROC AUC in 30 train-test splits do not cross the 0.6 border for the half of cancer types - breast (0.76), ovary, prostate, pancreatic, and uterus.

According to the mean lift of recall and mean lift of precision for the 0.05 probability percentile the most performant hotspots prediction model is for pancreatic cancer with the metric values around 10. Breast, liver and brain cancers belong to the next group by performance with 4.9-5.5 mean lift of recall while the lowest metric is observed for skin cancer (2.2).

All considered metrics are concordant in outlining the group of cancer types with the highest performance of hotspots prediction models (brain, breast, liver, pancreatic) as well as the worst performing model (skin cancer).

When comparing different hotspot labeling types prediction quality, one can see that for almost all cancer types the median test ROC AUC increases with an increase in threshold for hotspots labeling. In other words, it is easier to rank hotspots, which are more pronounced.

We compared prediction quality of breakpoint hotspots and individual breakpoints. As breakpoint prediction task is less class-imbalanced it implies a more narrow distribution of the test ROC AUC than for the hotspot prediction task. As it could be seen in Supplemental Table S9 the median value of the test ROC AUC for hotspot prediction models is significantly higher than for single breakpoints for all cancer types except for prostate cancer with almost equal values. Additionally, absolute values of the median test ROC AUC as well as the lift of recall/precision are quite low almost for all cancer types. The highest values of the median test ROC AUC are observed for the breast and prostate with 0.77 and 0.70 respectively while for other cancer types it is in the range 0.5-0.62. The mean lift of recall, higher than 1, is observed for the bone, brain and liver cancers at the 0.05 probability percentile. Thus, it could be seen that hotspots are better predicted than individual breakpoints.

Boruta feature selection

To determine important features for each type of cancer we used Boruta feature selection algorithm. Based on the best labeling type for each cancer type 10 random

initializations were performed for each of 30 train-test splits summing up to 300 total executions. Its description adapted to the specific research task is presented below.

For each train-test split of a dataset for a given cancer type:

0. Apply feature preprocessing. Define a final feature set as a set of all available features for a dataset.

1. For each random initialization (n = 10):
   1.1. For each iteration (k = 5):
      1.1.1. Create a set of “shadow” features by random row-wise permutation of all features in a final feature set.
      1.1.2. Train Random Forest on the final feature set extended with the set of shadow features, save mean decrease in accuracy and standard deviation for each used feature and calculate z-score as its ratio.
      1.1.3. Determine mzca - maximal z-score for the shadow features.
      1.1.4. Consider a real feature as important if its z-score is higher than mzca. Retain in the final feature set only those features that are recognized as important
      1.1.5. If the final feature set contains less than 5 features, the procedure is stopped and the next random initialization is started. Otherwise proceed with the next iteration.

As a result, we got the list of important features for each execution and transformed it into a heatmap of feature importance given in Supplemental Figure 6. Given the list, features for each cancer type were selected as the best if they were labelled as important in more than 50 from total 300 executions resulting in overall set of 46 features with the size of a feature set ranging from 4 to 21 for different cancer types (see Supplemental Table S13).

Then we built models to predict cancer hotspots by corresponding sets of important features. It was observed tha the quality of models in terms of lift of recall/lift of precision considerably decreased for pancreatic cancer and only slightly for breast and prostate cancer while for the remaining cancer types it insignificantly differed from the baseline. For the former cancer types it is essential to determine features that were not recognized as important in Boruta Feature Selection but nevertheless they

contributed to models quality in baseline models. In order to do this for a given cancer type, for each feature, not included in a set of important features, we estimated performance of a model trained on the set of the best features extended with this feature. As a result we found the sets of predictors for which the quality of the models was almost identical to baseline (in terms of lift of recall/lift of precision), but it was achieved using the best feature sets extended by the selected features for the three considered cancer types (see Supplemental Table S13). In particular, one feature was selected for pancreatic and prostate cancer while for breast cancer - two features. These predictors were combined with the best feature sets to compose the final feature set, which was used to train the next generation of models. The results are presented in Supplemental Table S15. For considered probability percentiles (0.03 and 0.05) the mean lift of recall and precision is significantly higher for models trained on the reduced feature sets compared to the sets of all features for three cancer types (blood, brain and liver) while for the remaining cancer types the difference is minor. In other words, a small set of important features can provide the quality comparable to baseline.

Random nature of cancer breakpoints

Earlier when comparing breakpoint hotspots and individual breakpoint prediction quality we formulated hypothesis that in general breakpoint locations are random but there are genome regions susceptible to breakpoints and identifiable by selected genomic features. To further investigate this question we created additional sets of target variables:

- we labeled new breakpoint hotspots using lower breakpoint density distribution percentiles: 75% / 90% / 95%. For some cancer types (bone, brain, liver, uterus) two neighboring percentiles in the set have the same value for threshold and hence same hotspot labeling. In these cases maximum of the two percentiles is shown in the research.
- we modified existing breakpoint hotspots labeling by exclusion of genomic windows without breakpoints

Based on new target variables we trained models using all available genome features and then compared their quality to hotspot prediction models also built on all features for each type of cancer.

Dependence of breakpoint hotspot prediction quality on labeling thresholds

Here we aimed at checking whether the quality of breakpoint hotspots will increase with an increase in labeling threshold or, in other words, is it true that the greater the number of breakpoints found in a window the more easily its location is explained by genome features.

Distributions of the test ROC AUC and lift of recall for 0.03, 0.05 and 0.1 probability percentiles by the labeling threshold and cancer type are depicted in Supplemental Figure 12; the summary statistics are shown in Supplemental Table S11. It could be seen that almost for all cancer types the higher the hotspots labeling threshold the higher is the shift of the distributions of quality metrics. The exceptions are bone and brain cancer; blood cancer also does not follow the trend of increasing lift of recall. It is worth to be noted that for more than the half of cancers (blood, breast, ovary, pancreatic, prostate and uterus) the distributions of the test ROC AUC for the highest hotspots labeling threshold (not less than 99%) do not intersect with the distributions for the 90% labeling type. Considering point estimates, the median test ROC AUC is given in Table 1 and the median test lift of recall – in Table 2. According to Table 2 the highest ratio of the median lifts of recall for the prediction of baseline hotspots (99%, 99.5%, 99.9%) and new considered hotspots (75%, 90%, 95%) for 0.03 probability percentile is observed for pancreatic, ovary, prostate and breast cancers (from 4,2 to 8,8 for 95%; from 4,8 to 10,7 for 90%; from 12,2 to 32,8 for 75%).

Thus, the higher the breakpoint density windows we aim to detect the higher the prediction quality, or in other words hotspots of a higher breakpoint density are more dissimilar to the other genomic regions.

The effect of removing windows without breakpoints

Next we investigated whether it is easier to identify hotspots in a set of all genome windows containing breakpoints than in the whole genome. Summary statistics for the experiment are given in Supplemental Table S10. When comparing test ROC AUC distributions for three types of models - hotspot vs all genome windows, breakpoints vs all genome windows, hotspots vs breakpoints - one can see that for liver, bone and uterus cancer baseline hotspots vs all prediction models outperform hotspot vs breakpoints prediction models (see Figure 5). For the remaining cancer types reducing a dataset to the breakpoint-containing windows does not significantly change the quality of example ranking.

To understand what is the relationship between different model performance for different types of cancer we calculated the median lift of recall μ and the lower bound of confidence interval for the mean lift of recall l for each model type. For each cancer type we compared two proportions: $\frac{l_{hotspots\ vs\ all}}{\mu_{breakpoints\ vs\ all}}$ is given in Supplemental Figure 14A and $\frac{l_{hotspots\ vs\ all}}{l_{hotspots\ vs\ breakpoints}}$ – in Supplemental Figure 14B. It could be seen that according to these figures all cancer types form two groups. Based on the conducted research these groups could be described as follows:

1. Pancreatic, breast, prostate and ovary cancers:
   - median lift of recall for breakpoints vs all models are close to zero so that breakpoints are hard to distinguish from the remaining genomic windows;
   - median lift of recall for hotspots vs all models are considerably high (4-18 times better selected comparing to random choice);
   - $\frac{l_{hotspots\ vs\ all}}{\mu_{breakpoints\ vs\ all}}$ is relatively high meaning that hotspots are easier to predict than breakpoints;
   - $\frac{l_{hotspots\ vs\ all}}{l_{hotspots\ vs\ breakpoints}}$ is close to one (ranges from 0.96 to 1.18) meaning that reducing the dataset to breakpoints-only windows does not complicate or ease the task of hotspot prediction;
   - these are top-4 cancer types by maximum number of breakpoints (from 71 000 to 192 000 points) with 7-12 different breakpoints variant types.

Based on these observations we could conclude that for these cancer types the hypothesis about random nature of breakpoints and recurrent nature of breakpoint hotspots, which differ (and hence could be distinguished by machine learning models) from other regions by considered genomic characteristics, is hard to reject.

2. The remaining cancers (brain, bone, blood, liver, skin, uterus):
   - median lift of recall for breakpoints vs all models are greater than those for cancer types of the first group (except skin cancer);

- median lift of recall for hotspots vs all models are relatively low in comparison with cancer types of the first group;
- $\frac{l_{hotspots\,vs\,all}}{\mu_{breakpoints\,vs\,all}}$ is relatively low (except for the uterus cancer) meaning that performance of hotspot prediction models do not significantly differ from the performance of breakpoint prediction models;
- $\frac{l_{hotspots\,vs\,all}}{l_{hotspots\,vs\,breakpoints}}$ is greater than 1.18 for 75% of cancer types (reducing dataset to windows containing breakpoints complicates hotspots prediction);
- 1500 - 55000 breakpoints are available for these cancer types with 4-7 different breakpoints variant types.

Statistics for this group of cancer types differ from those for the first group and for these cancer types the hypothesis is not confirmed. It is worth to be noted that taking into account relatively small breakpoints sample size this fact could be related to data insufficiency.

To summarize, we found 4 cancer types (pancreatic, prostate, breast and ovary) for which considered genomic features explain the most stable breakpoint locations and at the same time do not allow to distinguish between individual breakpoints and other regions. Firstly, it was revealed that for the majority of cancer types (all except bone, blood and brain) all performance metrics (ROC AUC, median lift of precision, median lift of recall) increase with an increase in hotspots labeling threshold meaning that hotspots of higher breakpoint density differ more from other regions than those of lower density. Secondly, as for these 4 cancer types breakpoints are poorly predicted while hotspots are predicted considerably better, approximately the same hotspot prediction quality, when compared to the remaining windows or only windows with breakpoints, lead to a conclusion that individual breakpoints hardly differ from other locations in terms of surrounding genome features.

References

1. International Cancer Genome Consortium (ICGC). Available from: https://icgc.org/.

2. Cheloshkina K, Poptsova M. Tissue-specific impact of stem-loops and quadruplexes on cancer breakpoints formation. BMC Cancer. 2019;19(1):434. doi: 10.1186/s12885-019-5653-x. PubMed PMID: 31077166; PubMed Central PMCID: PMCPMC6511154.
3. Davis CA, Hitz BC, Sloan CA, Chan ET, Davidson JM, Gabdank I, et al. The Encyclopedia of DNA elements (ENCODE): data portal update. Nucleic acids research. 2018;46(D1):D794-D801.
4. DNA PUNCTUATION [cited 2020 18.03.2020]. Available from: http://www.dnapunctuation.org/.
5. Cer RZ, Donohue DE, Mudunuri US, Temiz NA, Loss MA, Starner NJ, et al. Non-B DB v2. 0: a database of predicted non-B DNA-forming motifs and its associated tools. Nucleic acids research. 2012;41(D1):D94-D100.
6. Schmitt AD, Hu M, Jung I, Xu Z, Qiu Y, Tan CL, et al. A compendium of chromatin contact maps reveals spatially active regions in the human genome. Cell reports. 2016;17(8):2042-59.
