# Supplementary material for: Comprehensive analysis of cancer breakpoints reveals signatures of genetic and epigenetic contribution to cancer genome rearrangements
Source: PLoS Comput Biol. 2021 Mar 1;17(3):e1008749. doi: 10.1371/journal.pcbi.1008749 (PMC7951985; doi:10.1371/journal.pcbi.1008749)
Supplement: Attachment — Submitted filename: ResponsesReviewers.pdf [file pcbi.1008749.s040.pdf]

Dear Editor and reviewers,

Please find below our responses to reviewers' comments in blue.

Reviewer #1: Manuscript Number: PCOMPBIOL-D-20-00990

Title: Comprehensive analysis of cancer breakpoints reveals signatures of genetic and epigenetic contribution to cancer genome rearrangements

Authors: Cheloshkina and Poptsova

The authors established a predictive model by using a machine learning approach from a set of cancer breakpoint data and other genomic DNA feature datasets, such as non-B DNA, transcription factor binding sites, and epigenetic modifications. The model was evaluated by its power to predict genomic breakpoints in cancer and was used to estimate the contributions of these factors in chromosome breakage in cancer. The authors report here that although there are obvious cancer type-specific characteristics, non-B DNA and TF binding sites are the most powerful factors in predicting recurrent breakpoint hotpots in most cancer types.

This is a very timely study, aimed to address a very important question in the field: what are the reasons for chromosomal breakage hotspots in cancer? However, the impact of this study would be increased by addressing the following comments/concerns:

1. The appearance of mutations (breakpoints) in cancer genomes seems to be biased: some DNA breakage events might be lethal and therefore are eliminated from genomes quickly; some are associated with cancer development and therefore are enriched in cancer datasets. Although the authors mentioned "genomic positions (whole genes, exons, introns…)" at the beginning of the manuscript, and provided some supplementary data, this important feature was not adequately discussed. Did the authors detect any differences in genes vs. intergenic regions? Functions and importance of impacted genes? Transcribed regions vs. non-transcribed regions? Translated regions vs. untranslated regions? All of these issues should be addressed by the authors.

Indeed we did not discuss much this topic. Partially because we presented analysis of the distribution of breakpoints over genomic regions in our previous publication - https://bmccancer.biomedcentral.com/articles/10.1186/s12885-019-5653-x - see Figure 2, and the following text from this publication:

"We also checked the distribution of breakpoints and hotspots among different genomic regions, including whole genes, promoters, downstream regions, and the regions inside genes: 5' untranslated region (5' UTR), 3' untranslated region (3' UTR), coding exons and introns. Almost half of all breakpoints (48%) fall inside genes, though almost all are located inside introns (46%); 1.5% of breakpoints fall into coding regions, and less than 1 % fall into promoters, downstream regions or 5'UTR; 1.7% of all breakpoints fall into 3'UTR (Additional file 1: Figure S4). We checked the intersection of hotspots with whole genes and found that the percentage of breakpoint hotspots overlapping with genes varies from 10% in the uterus cancer to 40% in the blood and bone cancers (Fig. 2a). Overall the highest percentages of hotspots' overlap with whole genes are observed for the blood, bone, brain, and breast cancer (Fig. 2b). When looking at the distributions of all hotspots from all cancers stratified by the chromosomes, the chromosome 17 shows the maximum overlap (up to 30%) with the genes. The percentage of intersections of breakpoint hotspots with promoters, genes and downstream regions in different types of cancers and in different chromosomes is depicted in Fig. 3. For genomic regions the highest percentage is observed for

the breast, brain, blood, and bone cancers, and for chromosomes the highest percentage is observed for the chromosome 17 followed by the chromosomes 7 and 8."

In this resubmission we added an additional panel (C) to Fig 4. depicting individual contributions of each feature inside the genomic region group for breast cancer.

We updated results and discussion accordingly:

Importance of genomic regions, especially of 5'UTR and coding exons is definitely correlated with TFs and histone modifications that mark active transcription. Coupled with non-B DNA structures these results provide a hint on one of the major mutagenic process when breakpoints are generated as a result of RNA polymerase stalled on quadruplexes or stem-loop structures formed by repeats. The distribution of breakpoints and breakpoint hotspots (see Fig 2. and Fig.3 in [14]) showed that breakpoints are scattered all around genomes with probabilities of genomic region coverage. They are slightly underrepresented (48% vs 56%) in the gene bodies, though no significant enrichment/depletion is observed. Hotspots however underrepresented in gene bodies (only 10%), and in genes they are located mostly in introns. That is why the genomic region feature group did not appear as the top influential. GO enrichment analysis [31, 32] of genes harboring hotspots revealed significant enrichment in regulation cell cycle transitions, regulation of viral process, neurogenesis, phosphorylation and some other processes (see S12-14 Tables). For molecular function, enrichment is found for many types of binding – enzyme binding, RNA binding, nucleic acid binding, metal ion binding, organic cyclic compound binding. All together these findings support the idea that DNA breaks happen mostly during active genomic processes including replication..

In terms of transcribed and non-transcribed regions, unfortunately in this study we did not take into account RNA-seq data for cancer cells. Analysis of this data by itself would be an interesting study in the future. We also add a piece to the discussion after discussion of distant feature influence:

This fact could be explained from one side by the presence of active regulatory elements such as enhancers that are located at a long distance from a gene in DNA sequence but is found in close proximity in 3D chromatin organization. It is also indicative of transcriptionally active large-scale regions, and it will be a subject for further study to locate and analyze these regions from RNA-seq data from cancer patients.

2. The genomic features included in the study, i.e., non-B DNA, transcription factor binding sites, chromatin structures, and epigenetic modifications are somewhat associated with each other. For example, transcription occurs more frequently in open chromatin regions, which also have unique epigenetic modifications. And non-B DNA structures can affect all of these features. When evaluating each individual feature for its contribution to DNA breakage hotspots in cancer, how did the authors distinguish the impact of each feature? This needs to be addressed.

To distinguish between different feature groups influence on breakpoints hotspots prediction we build 7 models for each dataset – on each feature group separately. By comparing model performance of each group we assessed how much each feature group help to recognize breakpoint hotspots.

We added to the Results: "*We built machine learning models to investigate the impact of every feature group on prediction of breakpoint hotspots (S7 Table) by modeling breakpoints hotspots based on each feature group separately.*"

3. Maybe I have missed this information, but it needs to be clearly stated by the authors: were the data on transcription factor binding sites, chromatin organizations, and epigenetic modifications also from cancer

samples? If they were from healthy individuals, what was done to establish the connection to cancer? The authors should clearly address these points.

We realized that the journal does not impose restrictions on the article size and moved the entire section "Data" from Supplementary methods to Main methods. It should be clear now from where the data is taken from.

No, the data was not taken from cancer samples. It is an open data from Encode and Roadmap epigenomics and other public sources, and it is implied to be non-cancer genomes. It would be extremely interesting to analyze genomes together with the epigenetics data from cancer patients, we do not have this data available. To our knowledge, this data is not publicly available at the scale of our study of all cancer types.

Indeed, the epigenetic code also changes in cancer genomes, and it would be extremely interesting if there is an association between changes in epigenetic codes and breakpoint origination. That would be an interesting topic of a separate study – how changes in epigenetic markups are correlated with breakpoint formation. We would be happy to conduct such an analysis when we have the data.

Thank you for raising this issue. We added the paragraph to the Discussion:

It is important to note that the in this study we analyze non-cancerous distributions of features and cancerous breakpoints mapped to these distributions. In doing this we try to answer the questions – which regions of so-called normal genomes are more susceptible to breakpoint formation during cancerogenesis. Cancerous genomes undergo changes in epigenetics, and it would be of interest to investigate of how the changes in epigenetic landscapes are associated with breakpoint formation. The big data analysis retrieves the most important signals and reveals the major trends in the data (as non-B structures influence during transcriptional processes) however in the future when more data on single cancerous genome is available, one could extract more information.

4. Are there any "overlapped" regions identified by different features? Are these multiple feature regions more prone to breakage? The authors should address these questions.

To answer this question we conducted additional analysis and added the description of the results to the section "Contribution of each group of features on cancer breakpoint prediction":

We investigated whether the different feature group models identify the same or different breakpoint hotspots regions. For that we calculated mean Spearman correlation between predictions for each pair of feature groups (S10A Fig). The highest mean correlation over all cancer types is observed for histones modification-based models that are positively associated with TF- and methylation-based models (with correlation of 0.47 and 0.46 respectively). On the opposite, TAD-based models generate the most specific predictions with maximal correlation of 0.045 with TF-based predictions. Secondly, for each genome window in each test set we calculated the number of hotspots predicted with different number (1,2, or 3) of feature groups and analyzed the proportion of true hotspots. Comparison of median quality of the best feature group model (corresponds to label "1") and overlapped predictions of 2- or 3-feature group models demonstrates that for the majority of cancer types a single feature group model is more precise than the overlapped predictions (S10B Fig). For several cancer types (uterus, breast, bone, pancreatic) the median precision is only slightly higher with the increased standard deviation and the resulting number of identified hotspots (true positives) is lower. This fact, together with the observed uplift in combined models compared to the single feature group models, leads to a conclusion that in order to account for multiple factor contributions in cancer breakpoints hotspots' formation we need to use more complex model than the overlap between different one feature group models.

5. Line 202: why do some features exhibit "distant aggregation only"? For example, TF binding sites are in general pretty short, why do they affect only distal regions but do not affect nearby regions? Is it possible

that there are indirect associations? What are the consequences of TF binding, e.g., activation of transcription? The authors should discuss these points.

Added to the Discussion: "*This fact could be explained from one side by the presence of active regulatory elements such as enhancers that are located at a long distance from a gene in DNA sequence but is found in close proximity in 3D chromatin organization. It is also indicative of transcriptionally active large-scale regions, and it will be a subject for further study to locate and analyze these regions from RNA-seq data from cancer patients.*"

6. Can the authors provide information about the TF occupation at these sites in cancer cells?

Unfortunately, we do not have the data on TF in cancer cells available. There are studies (for example, Franco et al. Enhancer transcription reveals subtype-specific gene expression programs controlling breast cancer pathogenesis https://www.ncbi.nlm.nih.gov/pmc/articles/PMC5793780/) that provided the ChiP-seq data on histone modification in a particular type of cancer but these kind of data are not available for a large-scale studies. It would be a subject of a separate study to collect all the data available and superimpose with our findings. We address this issue now in the Discussion. (see Response to the comment 3 above)

7. When larger regions were included for long distance action, the "predictive power" was improved. Does that mean that this model could predict and identify more reported DNA breakage hotspots in cancer samples? How specific is the prediction? Did it also increase the rate of miss-hitting?

Yes, distant features also improve precision and recall. And we now mention this fact in the results:

Precision and recall metrics are also improved with an inclusion of distant features (results are not shown).

Below is the figure with a proof of this fact. We took one type of cancer (breast) and plotted the distribution of recall (A) and precision (B) for different feature sets for 99% labeling type. It could be seen that the addition of distant features to a set of local features lead to the evident increase in recall.

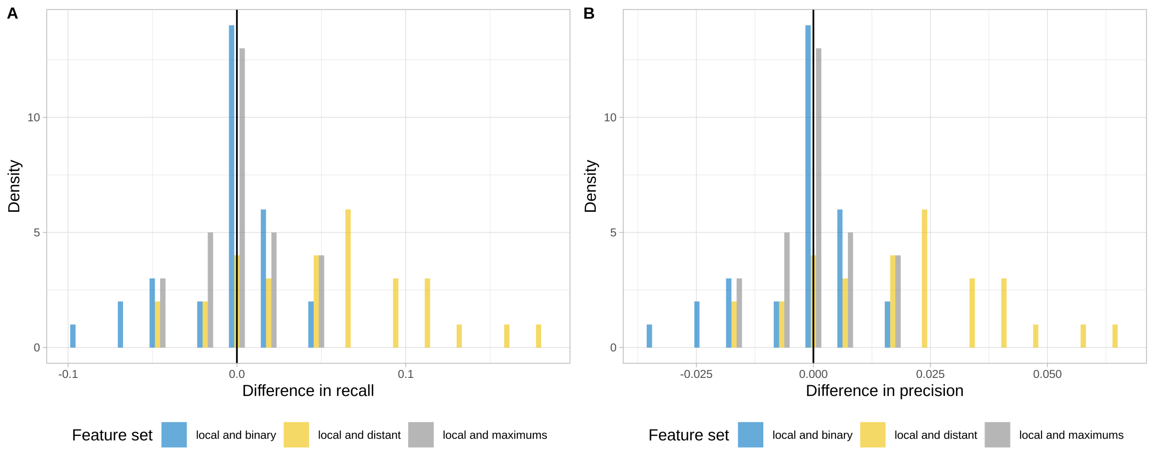

8. The fonts in the figures, particularly in Fig. 1, are too small to read.

Corrected: we increased font size in all figures.

Reviewer #2: The authors describe a comprehensive analysis of cancer breakpoints and their correlates with genomic and epigenetic features. The paper describes correlation levels of these features across cancer types, showing differences and similarities between cancer types. It is however not clear how new these insights are compared to previous models/analysis (ref 9-14 in the introduction).

Summary of all mentioned cancer-related studies together with their prediction quality is given now in S1 Table.

Perhaps the most interesting insight from the paper is the potential difference between breakpoint hotspots and singletons. However, this aspect is not analysed or described in-depth, and could have been a greater focus of the paper.

Indeed, the topic is very interesting, and we are thinking to continue working on it further. But we think it will comprise a subject for a separate study – to see which cancer genome have a higher degree of randomness in terms of breakpoint prediction.

We added more to the discussion: "*It will be of interest to further research to investigate, which cancer genomes have a higher degree of randomness in terms of breakpoint prediction, and what are the major mutagenic processes for those breakpoints that can be predicted.*"

Furthermore, I have multiple concerns regarding the use of test sets, accuracy metrics for imbalanced data, and benchmarking with other published models/methods.

Not clear if test set is independent
It is not clear if the test sets are completely independent of the training sets. The authors should avoid information leakage between training and test sets. Furthermore, for reproducibly and subsequent model benchmarking/comparison by other groups, the authors should keep a separate withheld test set for reporting of AUC/AUPRC measures. The accuracy (AUPRC/AUC) in figures should be mainly derived from this test set. All data (breakpoints, cancer types, genomic coordinates) for this test set should be included in the supplement.

In this study we used 30-times repeated train-test (70/30) splits with test set being independent. This resampling scheme was selected due to a high class imbalance, which causes quality measurements vary from one test set to another. To get a reliable estimate of quality we measure all metrics on test set of each of 30 splits (while training models on corresponding train set of particular split) and report mean or median of these 30 measurements.

This procedure is described in section Methods: "*To get reliable model performance estimates we used 30-times repeated and stratified train-test split with 30% of data in test sample with splits stratified by chromosome and position inside it. The quality of models was measured <u>on each of 30 test sets</u> with ROC AUC and lift of recall/lift of precision metrics (the number of times recall/precision of the model is greater than a random choice using specific probability percentile as a threshold).*"

The scripts and final datasets for this resampling scheme is available on Github (https://github.com/KseniiaCheloshkina/cancer_breakpoints_hotspots_prediction_wide)

There are totally 104 datasets of a total size of 5Gb, so that we cannot upload them as a Supplementary. They are all available at github.

Accuracy metrics and issues of class imbalance

The authors use AUC for assessing model accuracy. However, its not clear how the negative set was defined and how imbalanced the positive and negative sets are. This should be described in the methods.

We describe division into positive and negative classes in Methods: "*The window was designated as a hotspot if its breakpoint density is greater than the specified density distribution percentile (99%, 99.5%, 99.9%)"*

Hence, we considered 3 types of target labels: with class imbalance 99% of negatives vs 1% of positives, 99.5% of negatives vs 0.5% of positives, 99.9% of negatives vs 0.1% of positives.

In their scenario, true negatives are likely arbitrary and greatly dominate the positive class. A more suitable accuracy metric in this setting is the area under the precision-recall curve (AUPRC). The authors should report AUPRC (in addition to, or instead of AUC) numbers throughout text and figures.

We agree with the reviewer that AUCPR is often used in cases of imbalanced classes. In our case its use is inappropriate since AUCPR depends on the proportion of positive examples in a dataset and that makes it impossible to compare datasets with different class balance (referred to as "labeling types" in the paper). In the section "Contribution of each group of features on cancer breakpoint prediction" we consider 3 labeling types (99%, 99.5%, 99.9%) for breakpoint hotspots classification task while in the section "Non-predictable individual breakpoints and highly predictable breakpoint hotspots" we additionally consider 75%, 90%, and 95% class divisions.

That is why, as a substitution for AUCPR, we report the lift of precision and lift of recall, which are related to AUCPR.

R-squared not reported
To get a sense of how much of the variance these top-features capture, alone and in combination, the authors should also report measures of R-squared.

R-squared is not reported because of model specification: we predict binary labels of breakpoint/breakpoints hotspot presence in a genomic window, we can only measure binary classification metrics, not the regression one.

Lack of clearly defined negative set
It is not clear how the negative set (regions without breakpoints) was defined for model training and testing.

This is described in Methods: "*The window was designated as a hotspot if its breakpoint density is greater than the specified density distribution percentile (99%, 99.5%, 99.9%) for corresponding cancer type. These three thresholds are referred to as labeling types. Additionally, binary labels were assigned to genome windows indicating presence/absence of breakpoints.*"

More detailed description was in Supplementary Material earlier. Now we moved it into the main Methods: "*For each window, located in a specific chromosome, breakpoint density was calculated as a number of breakpoints overlapping with the window divided by the total number of breakpoints in the chromosome separately for each cancer type. Given breakpoint density we highlighted hotspots - regions of the genome significantly enriched with breakpoints. Three percentiles - 99%, 99.5% and 99.9% - of breakpoint density distribution were calculated for each cancer type and used as a threshold for labeling hotspots. Total number of breakpoints sorted by cancer type and labeling is given in Supplemental Table S4. Additionally, binary labels were assigned to genome windows indicating presence/absence of breakpoints.*"

No comparison with previous studies/methods
The authors cite several previous studies analyzing associations between cancer breakpoints and epigenetic

features. The authors should compare their models performance with relevant existing approaches/models/methods (e.g. references 9-14 in the paper).

We summarized all the reviewed material in one table (new Supplementary Table S1) with benchmark of considered approaches for modeling and analysis of point and breakpoint mutations.

Recent studies used different methods for analysis. In our research we used predictive modeling with the help of machine learning while earlier works used statistical testing. Other researchers, who applied machine learning methods, aimed at prediction of breakpoints density and hence performed regression task which is incomparable with classification task we tried to solve. Our previous research (Cheloshkina and Poptsova, 2019 https://bmccancer.biomedcentral.com/articles/10.1186/s12885-019-5653-x) limited to investigation of stem-loops and quadruplexes is the only classification models for comparison.

Concern that non-B DNA features are enriched for repeat regions
It is concerning that the non-B DNA feature group is largely composed of regions with repetitive sequences (e.g. Figure 2). This may cause mapping/alignment errors and affect breakpoint calling, i.e. a higher false-positive rate in repeat regions. The authors should consider the align/map-ability of these regions, and evaluate the impact/bias this has on this feature group. The authors should remove/filter all regions with low mappability from the analysis.

We filtered out these windows from the analysis.

In the Methods we mentioned that data were preprocessed: "*For breakpoint hotspot analysis we split the genome in 100 kb non-overlapping windows, calculated cancer breakpoints density and removed unstable regions*"

More detailed description was in Supplementary Methods, which we moved now to the main Methods: "*To control for the genome instability introduced by natural topological constrains some windows were removed. Precisely, we excluded windows intersected with centromeres and telomeres, the first and last window of each chromosome. Additionally we took into consideration the DAC blacklist (https://www.encodeproject.org/annotations/ENCSR636HFF/) comprising a set of genome regions recognized as low mappable or anomalous and recommended to be removed from genome studies. Concerning blacklisted regions it is worth to be noted that the percent of breakpoints laying in these regions is very small. So it is minimal for liver cancer with 0.09% of breakpoints located in the regions, maximal for brain cancer (6,8%) and is in the range 0.4% - 1.5% for other cancer types. Finally, Y and MT chromosomes were not included in the research as they contain relatively small number of breakpoints. Finally we selected 29288 100-kb windows.*"

Minor comments

* Missing figure caption/text for Figure 2.

Added caption: "***Fig 2. Boruta selected feature sets by cancer type and feature group.*** *The most important features for all cancer types selected by Boruta feature selection method for each cancer type separately.*"

* P. 7 "When analyzing group feature importance for all cancers combined, the non-B DNA structures are on the first place followed by transcription factors and genomic regions (2A and 2B Fig)". It is not clear from figure 2 that anything is in 1st or 2nd place. This should be quantified and explained better. Also, Figure 2 caption text is missing.

We added mean values to the text (section Results): "*When analyzing group feature importance for all cancers combined, the non-B DNA structures are on the first place followed by transcription factors and genomic regions (2A and 2B Fig) <u>with mean lift of recall (3.18 and 3.02) and mean ROC AUC (0.66 and</u>*

0.62) slightly higher for non-B DNA than TF"

* P. 8 ""Independent from the methods – group or Boruta feature importance – two best performing feature groups were always non-B DNA and TFs." How is this conclusion reached? This is also a key claim in the abstract. The previous paragraph refers to many different supplementary figures, but the authors do not provide specific and direct data (e.g. a statistical test) in a main figure to support this claim.

To reach this claim we considered the next machine learning procedures:

1) We built models on each feature group separately and ranked models by performance. Based on lift of recall "*The results show that the best feature group significantly (by 0.25) outperforms others for almost all cancer types and even by 0.5 for 3 cancers (blood, pancreatic and prostate). This outperforming group included TFs for 5 cancers (liver, skin, prostate, ovary, breast), non-B DNA structures for 2 cancers (brain and bone), genomic regions (pancreatic), HMs (blood), and HDNase (blood). Non-B DNA structures are always included into the top-3 important groups for all types of cancers, TFs are present as the top-3 in 8 types of cancers (except for blood and brain), genomic regions in 5, and HMs in 4 cancer types.*"
2) We used Boruta Feature Selection method to recognize the most important features from all the available features: "*Additionally, we investigated the influence of individual features with the Boruta feature selection method (see Methods) considering individual contribution of every feature from every group (S6 Fig, S8 Table). Totally, the method selected 50 important features with 5 - 23 features per cancer type (Fig 2). Similar to the feature group importance results, presented earlier, the Boruta selection method also highlighted features mostly from non-B DNA, TFs and genomic regions groups (at least one feature of each group is included in a set of best features for 8 cancer types).*"

Both considered procedures agree in dominating role of non-B DNA and TFs.

* Figure 4: It is difficult to evaluate the difference between local and distant features in these plots since the importance axis have different scales. Its also not clear if local and distant features were evaluated alone or in combination. The authors should run the importance analysis using _all_ features (both local and distant) and plot them both local/distant features in the same plot.

In the study we used both local and distant features (Results, section "Inclusion of distant features improves prediction power of the models"): "*Based on these results we included distant features in the overall feature set (designated with the prefix "upper_")*". For better plot comprehension we set the same scale for these features in Figure 4.

* P. 11: "Previous attempts to build models using full breakpoints distribution did not achieve good prediction performance." Which attempts were these? Please explain.

In this sentence we are making reference to the Introduction section: "*Different studies showed enrichment of cancer breakpoints regions with non-B DNA structures [9-13] however predicting breakpoint densities with the same set of non-B DNA structures and epigenetic factors as for the cancer point mutations could achieve R2 of 10% only for all cancers with an exception of 18% for the breast cancer [9]*"

We added citation and exact values of model performance: " *Previous attempts to build models using full breakpoints distribution did not achieve good prediction performance (regression task, R2 of 10% for all cancers except for 18% for the breast cancer [9]).*"

* Figure 5: " The results are presented for 0.03, 0.05 and 0.1 probability percentiles." It is not clear what the x-axis in figure 5 denotes.

A) "The results are presented for 0.03, 0.05 and 0.1 probability percentiles."
In Figure 5B each panel refers to the probability percentile (0.03/0.05/0.1). The term "probability percentile" is explained in S1 File section Evaluation metrics. We added to Methods section short explanation: "*The quality of models was measured on each of 30 test sets with ROC AUC and lift of recall/lift of precision metrics. These metrics show the number of times recall/precision of the model is greater than a random choice using specific probability percentile as a threshold where each probability percentile is given by the percentile of the model output probability distribution. In particular, the lift of recall for a specific probability threshold (given by a percentile of a probability distribution) is calculated as the recall divided by this percentile of the probability distribution. For example, since it is supposed that in case of a random choice recall approximates the probability percentile (labeling n% of examples gives n% recall), by dividing the model recall by the probability percentile we get the number of times a model is better compared to a random choice.*"

B) In x-axis of Figure 5 we plot labeling type.
The definition is given in Methods: "*The window was designated as a hotspot if its breakpoint density is greater than the specified density distribution percentile (99%, 99.5%, 99.9%) for corresponding cancer type. These three thresholds are referred to as labeling types (labeling thresholds).*" as well as in Methods section Data (earlier S1 File section Data): "*Given breakpoint density we highlighted hotspots - regions of the genome significantly enriched with breakpoints. Three percentiles - 99%, 99.5% and 99.9% - of breakpoint density distribution were calculated for each cancer type and used as a threshold for labeling hotspots.*"
