# Supplementary material for: Comprehensive analysis of cancer breakpoints reveals signatures of genetic and epigenetic contribution to cancer genome rearrangements
Source: PLoS Comput Biol. 2021 Mar 1;17(3):e1008749. doi: 10.1371/journal.pcbi.1008749 (PMC7951985; doi:10.1371/journal.pcbi.1008749)
Supplement: Attachment — Submitted filename: ResponsesToReviewers291220.pdf [file pcbi.1008749.s042.pdf]

Reviewer #2: “Selecting the most important features at the individual level of cancer genome, for the first time we could build the model that outperforms all previous machine learning models for cancer breakpoint predictions”

###

Authors: “We included PR AUC values and updated the corresponding tables and figures: Figure S15 and Table S18 – combined models on full feature set for hotspots; ...”

I appreciate the extra work the authors did to include precision and recall for their models and features. My main concern is now that these numbers are hidden away in supplemental tables, and nowhere in the manuscript is it mentioned that their models are only achieving 2-5% precision and recall <40%, resulting in an AUCPR <0.02 (e.g. Fig S15). Overall, this suggest that their models are good at predicting regions where there are NO breakpoints (negative class) but very poor at predicting actual breakpoint regions (the very rare positive class), resulting in high AUCs and very low AUCPRs. This is a classical machine learning problem and pitfall with class-imbalanced data. Based on the newly reported precision/recall data, I am concerned that this study will severely mislead the field with a claim that cancer breakpoints can be be robustly predicted from genomic/epigenomic features:

Abstract: "predictive models for cancer breakpoint formation achieved 70-90% ROC AUC for different types of cancers."
Introduction: “... for the first time we could build the model that outperforms all previous machine learning models for cancer breakpoint predictions”

I would recommend that the authors rework their manuscript and figures with a fair, unbiased and open discussion of the low precision and AUCPR.

Indeed, the precision and recall are low that is why we used lift of precision and lift of recall. Since the main focus of the paper is to examine contribution of the features to the predictive power of the model and to verify the idea that the omics approach helps to improve ML models’ prediction power, we did not emphasize the low precision and recall. But we agree it would be fair to discuss it, and now we corrected the Abstract, emphasized it in the Results and included the section in the Discussion.

The main Figure 3 and Figures S9, S15 that compare relative performance of each model has PR AUC. For the other figures, as we now explain at the beginning of the Results, we compare model performance with ROC AUC and lift of recall.

GitHub repository contains all the code for this study: https://github.com/KseniiaCheloshkina/cancer_breakpoints_hotspots_prediction_wide. The folder "analytics" contains all the code (in Rmarkdown files) needed to reproduce main figures. To make it easier to navigate through the data, we added notebook "plot_main_figures" to the same folder. It uses data from "datasets/output_for_figures/" to plot the final figures of the manuscript.
