# Supplementary material for: Comprehensive analysis of cancer breakpoints reveals signatures of genetic and epigenetic contribution to cancer genome rearrangements
Source: PLoS Comput Biol. 2021 Mar 1;17(3):e1008749. doi: 10.1371/journal.pcbi.1008749 (PMC7951985; doi:10.1371/journal.pcbi.1008749)
Supplement: Attachment — Submitted filename: ResponsesReviewers 221120.docx [file pcbi.1008749.s041.docx]

**Comments to the Authors:**

Reviewer #2:>> Reviewer: “The authors should report AUPRC (in addition to, or instead of AUC) numbers throughout text and figures.”
>> Authors: “We agree with the reviewer that AUCPR is often used in cases of imbalanced classes. In our case its use is inappropriate since AUCPR depends on the proportion of positive examples in a dataset and that makes it impossible to compare datasets with different class balance”

The authors attempt to discuss their way through this point. To avoid misleading the readers, they MUST report AUPRC for one of these splits, for example the middle split (1% positives). The authors can use the other reporting metrics when they compare across splits containing different positive proportions.

We included PR AUC values and updated the corresponding tables and figures:

Figure S15 and Table S18 – combined models on full feature set for hotspots;

Table S10 – combined models on full feature set for breakpoints;

Table S8 – combined models on the best feature set;

Table S7 – single feature group models;

Figure 3, Figure S9 – comparison of single feature group models and combined models on the best feature set.

We added to the Results, the section “Contribution of each group of features on cancer breakpoint prediction”:

We also report PR AUC as specifically informative for class-imbalance problems (Fig 3, S9 Fig, S8 Table). As we can see from Fig.3, according to PR AUC, the top three feature groups coincide with those determined according ROC AUC or lift of recall for all types of cancer (except for the blood cancer for 99% labeling type), and the ranking of cancer types according to the model performances is generally preserved among three metrics.

>> Reviewer: “To get a sense of how much of the variance these top-features capture, alone and in combination, the authors should also report measures of R-squared.”
>> Authors: “R-squared is not reported because of model specification: we predict binary labels of breakpoint/breakpoints hotspot presence in a genomic window, we can only measure binary classification metrics, not the regression one.”

The authors are not providing any help here. As a reader, I have no sense of how well these individual covariates explain the variance in the data. Do they explain 0.01% or 50% better than a naive/null model? Lift of recall, feature importance, and AUC (see above) are not intuitive metrics. There are interpretable metrics for binary data, such as McFadden’s R2 (“<https://en.wikipedia.org/wiki/Logistic_regression#Evaluating_goodness_of_fit>”). The authors should plot McFadden’s R2 for these top features.

We re-run our models using logistic regression and presented results in S10 Fig and S9 Table.

We added to the Results, the section “Contribution of each group of features on cancer breakpoint prediction”:

To evaluate goodness-of-fit of the all-features and single feature group models, we calculated McFadden’s R-squared (pseudo R-squared) for 99% and 99.5% labeling type (S10 Fig, S9 Table) by building logistic regression models. We found that for the half of cancers (breast, ovary, pancreatic, skin, and blood) non-B DNA based models produced the maximal pseudo R-squared while for the rest the all-features model performed the best. In absolute values, the maximal McFadden’s R-squared over all cancer types are observed for breast, prostate and ovary cancers with 0.087, 0.066 and 0.061 respectively.

>> Reviewer: “The authors should compare their models performance with relevant existing approaches/models/methods (e.g. references 9-14 in the paper).”
>> Author: “We summarized all the reviewed material in one table (new Supplementary Table S1) with benchmark of considered approaches for modeling and analysis of point and breakpoint mutations. Other researchers, who applied machine learning methods, aimed at prediction of breakpoints density and hence performed regression task which is incomparable with classification task we tried to solve. Our previous research … is the only classification models for comparison.”

Table S1 is a simple summary of existing methods with accuracy statistics copied from the respective papers. There is NO direct comparison/benchmark with the authors method on the same datasets. Firstly, while previous studies may have used regression to predict (continuous) breakpoint density, the authors have instead used (ad-hoc) discretisation of the density (using various cutoffs), and they therefore claim methods/results cannot be compared. This is NOT the case. The authors can run existing (regression-based) methods, set a cutoff for the prediction score to obtain positive and negative classes, and then compare with their own model. It is important that the authors compare their method to existing approaches so the reader can gauge the improvement above current state of the art.

We obtained the data used in Georgakopoulos-Soare et al. from the authors and built regression models using only non-B and histone modifications (as the authors presented in Georgakopoulos-Soare et al.), and then we built the regression models using all our features. With these experiments on the Georgakopoulos-Soare et al. data set we demonstrated that omics approach is more powerful. We also converted the regression task to classification, as the reviewer suggested, by setting 99% percentile threshold for breakpoint hotspots and performed the same modeling as we did for our data. For that we reported all three metrics of model performance: ROC AUC, PR AUC and lift of recall.

In the benchmark figure we could not plot boxplots as in the original paper of Georgakopoulos-Soare et al. because we do not have the data on the authors’ model performance, we only have the authors’ raw data on breakpoints. Instead we plotted the median R2 from the Supplementary Figure 8 of Georgakopoulos-Soare et al. publication.

We added the new section to the Results: “Comparison with the state-of the art approach” describing this new benchmark analysis – highlighted in yellow in the main text.

>> Reviewer: “It is concerning that the non-B DNA feature group is largely composed of regions with repetitive sequences (e.g. Figure 2). This may cause mapping/alignment errors and affect breakpoint calling, i.e. a higher false- positive rate in repeat regions.”
>> Authors: “More detailed description was in Supplementary Methods, which we moved now to the main Methods… Additionally we took into consideration the DAC blacklist (<https://www.encodeproject.org/annotations/ENCSR636HFF/>) comprising a set of genome regions recognized as low mappable or anomalous and recommended to be removed from genome studies.“

The authors did no extra work to convince me this is not an issue. The encode blacklist is a very small and conservative list of regions. The authors could have analyzed/filtered the data with the mappability tracks from UCSC genome browser (e.g. “<https://genome.ucsc.edu/cgi-bin/hgFileUi?db=hg19&g=wgEncodeMapability>”), and showed me this had no effect on their conclusions about non B-DNA features. Their decision not do this makes we worry about the robustness of their conclusions.

Indeed, mapping of repeats is the major issue that has been addressed in many studies calling structural variants. Since we took the data from the ICGC we initially relied on their structural variant pipelines.

To be exact, we used the release 28 from ICGC (March 27^th^ 2019). The pipelines for Structural Variant (SV) calling, described in the PanCancer Nature special issue publication (Consortium 2020), take into account all the available mappability tracks for the final SV calling - Gencode Mappability track, UCSC High Seq Depth track, UCSC Simple-Tandem repeats, UCSC Repeat-Masker, DUKE-Excluded, DAC- Blacklist, UCSC Selfchain.

In this resubmission, we verified how many breakpoints, which we downloaded after the above-mentioned pipe-line processing, fell into low mappability regions. We took Duke 35-mer track with mappability less than 0.5, and found that approximately ~8-13% of all breakpoints fell into those regions (Supplementary Table 16). Taking breast cancer data as a case study, we excluded these breakpoints falling into low mappable regions, re-run our models on the filtered data set, and applied Boruta feature importance procedure. The results are presented in Supplementary Figure 14, and they showed that non-B structures (comprising not only repeats but also G-quadruplexes and Z-DNA) are still in the top 10 list.

It seems that the obtained results are in line with the latest studies verifying that CNVs are enriched in low mappability regions and repeats (Monlong, Cossette et al. 2018) and It is now confirmed by long-read sequencing methods that SV is often fall in repeat regions (Sulovari, Li et al. 2019). In (Monlong, Cossette et al. 2018) the authors “identified 3455 novel regions with CNVs in more than 1% of the population. 81.3% of these regions were located in low-mappability regions while 18.4% were located in extremely low-mappability regions.” This finding tells us that in our study, due to filtering procedures in SV calling pipelines, we underestimate the proportion of breakpoints generated by repeat instabilities. Nevertheless machine learning approach captured this trait.

Thank you for raising this issue. We think it is important to highlight it in the paper. We added the corresponding text to the Discussion (highlighted in yellow).

Repeats are difficult to map and though structural variant calling pipelines take into account information on low mappability regions, we made an additional analysis by filtering breakpoint data with low mappibility regions. We used 35-mer Duke Uniqueness track from UCSC genome browser (<https://genome.ucsc.edu/cgi-bin/hgFileUi?db=hg19&g=wgEncodeMapability>) and found that 8-13% of breakpoints falls into low mappable regions - regions with less than 0.5 uniqueness score (S16 Table). Additionally, on example of breast cancer we conducted the entire analysis for the filtered data set (S17 Table). Boruta feature selection method confirmed non-B DNA feature group as the most important with repeats remaining in top-10 list following quadruplexes and preceding Z-DNA (S14 Fig). The latest studies confirm that CNVs are enriched in low mappability regions and repeats [22] and the fact that SVs are often fall in repeat regions, is now verified by long-read sequencing methods [23]. These findings suggest that in our study, due to filtering procedures in SV calling pipelines, we underestimate the proportion of breakpoints associated with repeats.

Consortium, I. T. P.-C. A. o. W. G. (2020). "Pan-cancer analysis of whole genomes." Nature **578**(7793): 82-93.

Monlong, J., P. Cossette, C. Meloche, G. Rouleau, S. L. Girard and G. Bourque (2018). "Human copy number variants are enriched in regions of low mappability." Nucleic Acids Res **46**(14): 7236-7249.

Sulovari, A., R. Li, P. A. Audano, D. Porubsky, M. R. Vollger, G. A. Logsdon, C. Human Genome Structural Variation, W. C. Warren, A. A. Pollen, M. J. P. Chaisson and E. E. Eichler (2019). "Human-specific tandem repeat expansion and differential gene expression during primate evolution." Proc Natl Acad Sci U S A **116**(46): 23243-23253.
