# Supplementary material for: Comprehensive analysis of cancer breakpoints reveals signatures of genetic and epigenetic contribution to cancer genome rearrangements
Source: PLoS Comput Biol. 2021 Mar 1;17(3):e1008749. doi: 10.1371/journal.pcbi.1008749 (PMC7951985; doi:10.1371/journal.pcbi.1008749)
Supplement: Attachment — Submitted filename: ResponseToReviewers.docx [file pcbi.1008749.s043.docx]

The manuscript has been revised by a native proofreader. We provide a version showing the corrections.
